# Transgenic refractory *Aedes aegypti* lines are resistant to multiple serotypes of dengue virus

**DOI:** 10.1038/s41598-021-03229-4

**Published:** 2021-12-13

**Authors:** Wei-Liang Liu, Chia-Wei Hsu, Shih-Peng Chan, Pei-Shi Yen, Matthew P. Su, Jian-Chiuan Li, Hsing-Han Li, Lie Cheng, Cheng-Kang Tang, Shih-Hsun Ko, Huai-Kuang Tsai, Zing Tsung-Yeh Tsai, Omar S. Akbari, Anna-Bella Failloux, Chun-Hong Chen

**Affiliations:** 1 National Mosquito-Borne Diseases Control Research Center, National Health Research Institutes, Miaoli, Taiwan; 2 Institutes of Molecular and Cellular Biology, National Taiwan University, Taipei, Taiwan; 3 Graduate Institute of Microbiology, College of Medicine, National Taiwan University, Taipei, Taiwan; 4 Unit of Arboviruses and Insect Vectors, Department of Virology, Institute Pasteur, Paris, France; 5 Department of Biological Science, Nagoya University, Nagoya, Japan; 6 Institute for Advanced Research, Nagoya University, Nagoya, Japan; 7 National Institute of Infectious Diseases and Vaccinology, National Health Research Institutes, Miaoli, Taiwan; 8 Institute of Information Science, Academia Sinica, Taipei, Taiwan; 9 Section of Cell and Developmental Biology, Division of Biological Sciences, University of California, San Diego, La Jolla, CA 92093 USA; 10 National Institute of Infectious Diseases and Vaccinology, National Mosquito-Borne Diseases Control Research Center, National Health Research Institutes, Zhunan, Taiwan

**Keywords:** RNAi, Pathogenesis

## Abstract

The areas where dengue virus (DENV) is endemic have expanded rapidly, driven in part by the global spread of *Aedes* species, which act as disease vectors. DENV replicates in the mosquito midgut and is disseminated to the mosquito’s salivary glands for amplification. Thus, blocking virus infection or replication in the tissues of the mosquito may be a viable strategy for reducing the incidence of DENV transmission to humans. Here we used the mariner *Mos*1 transposase to create an *Aedes aegypti* line that expresses virus-specific miRNA hairpins capable of blocking DENV replication. These microRNA are driven by the blood-meal-inducible carboxypeptidase A promoter or by the polyubiquitin promoter. The transgenic mosquitoes exhibited significantly lower infection rates and viral titers for most DENV serotypes 7 days after receiving an infectious blood meal. The treatment was also effective at day 14 post infection after a second blood meal had been administered. In viral transmission assay, we found there was significantly reduced transmission in these lines. These transgenic mosquitoes were effective in silencing most of the DENV genome; such an approach may be employed to control a dengue fever epidemic.

## Introduction

Dengue fever is a major mosquito-borne viral disease affecting some parts of the world. The World Health Organization has estimated that 50 million cases of dengue virus (DENV) infection occur worldwide per year, and that over 2.5 billion people are now at risk of infection^1–3^. Dengue fever is caused by DENV, with four closely related yet antigenically distinct serotypes (DENV-1, DENV-2, DENV-3, and DENV-4)^4,5^. This serotype diversity hampers the prevention of dengue fever and creates difficulties for producing a single effective vaccine. The four serotypes differ from each other by 25–40% at the amino acid level. Moreover, the DENV-1 to -4 serotypes comprise multiple genotypes. This diversity makes it particularly difficult to develop effective anti-DENV treatment methods.

DENV is transmitted by female mosquitoes mainly of the *Aedes aegypti* and *Ae. albopictus* species. Once mosquito acquired the DENV, virus would infect midgut in 3–5 days, disseminate into circulatory system to reach salivary glands in 7–10 days, and transmit virus to next host in 10–14 days^6–8^. Therefore, blocking virus acquisition or replication in the tissues of the mosquito may be a strategy for reducing the incidence of DENV transmission to humans. Several approaches can be employed to control mosquito-borne diseases, for example, using chemical pesticides, releasing *Wolbachia*-infected mosquitoes^9^, and using genetic engineering for mosquito control. Nevertheless, blocking virus acquisition or replication in the tissues of the mosquito may be more efficient, and could be a crucial strategy for reducing the incidence of DENV transmission to humans. Studies have revealed that a gene drive to carry a refractory gene into a population is now feasible in some cases^10–12^. Driving the anti-DENV gene into the mosquito population is thus also an option for DENV control. Such effector genes can help prevent the relevant disease, and gene-drive technology can be used to drive the introgression of these genes into a population of *Ae. aegypti*, replacing the disease-competent population with a refractory one.

In recent years, more promising genetic-based strategies have been developed to control mosquito populations^13–16^. Among them, RNA interference (RNAi) has been shown to be effective against mosquito-borne diseases, including DENV, chikungunya virus (CHIKV), and Zika virus (ZIKV)^17–19^. RNAi can be triggered by conventional inverted repeats or synthetic microRNAs (miRNAs)^20,21^. miRNAs are 20 to 24 nucleotides (nt) small RNAs that regulates many functions, such as gene expression, post-transcriptionally through translational repression and mRNA cleavage. In the miRNA processing pathway, the miRNA precursors (pri-miRNA) are processed into ~ 70 nt hairpins by Drosha and folded into stem-loop structures in the nucleus. They are subsequently exported into the cytoplasm by Exportin5. In the cytoplasm, the stem-loop structures are cleaved into ~ 22 nt miRNA duplexes by Dicer-1, which are then loaded into either Ago-1 or Ago-2 proteins in miRNA-induced silencing complexes (RISCs) according to their different structural properties^22–24^. By recognizing a complementary sequence contained within a target RNA, RISCs execute silencing through RNA degradation, translational inhibition, or both methods^25,26^. To construct the artificial antiviral synthetic miRNA, the consensus sequence region of the virus was selected as the target site for the antiviral miRNA^27,28^. The corresponding miRNAs were designed and cloned in tandem to make a miRNA-based RNAi cassette. Using a miRNA-based genetically engineered model to produce a polycistronic artificial miRNA permits the simultaneous expression of multiple synthetic miRNAs that mediate the degradation of various regions of one target RNA sequence or multiple RNA sequences from different DENV genes.

Transgenic mosquitoes also require functional promoters to drive expression of the synthetic miRNAs following a blood meal (BM). Active promoters have been identified in the cells of the *Ae. aegypti* mosquito. The midgut is reportedly the primary tissue for DENV replication. Both the polyubiquitin (*PUB*) promote^27^ and the blood-meal-inducible carboxypeptidase A (*CPA*) promoter^28^ are active in the midgut epithelial cells of *Ae. aegypti*. Moreover, multiple blood feedings in female *Ae. aegypti* during a single gonotrophic cycle are associated with variation in the pattern of DENV transmission^29^. Therefore, whether multiple blood feeds may lead to increases in density or associated changes in DENV blocking ability of refractory transgenic mosquitoes should be investigated.

Here, we demonstrate anti-DENV miRNAs under the control of *AeCPA* and *AePUB* promoters that drive the expression of a polycistronic cluster of eight synthetic miRNAs; these miRNAs were engineered to target conserved sequences in the four DENV genomes. Our experiments revealed that our transgenic mosquitoes had an effective antiviral capacity. The antiviral capability of transgenic lines may present new opportunities for DENV control.

## Results

### Designing the anti-DENV miRNA and targeting sites on the DENV genome

**Figure 1:**
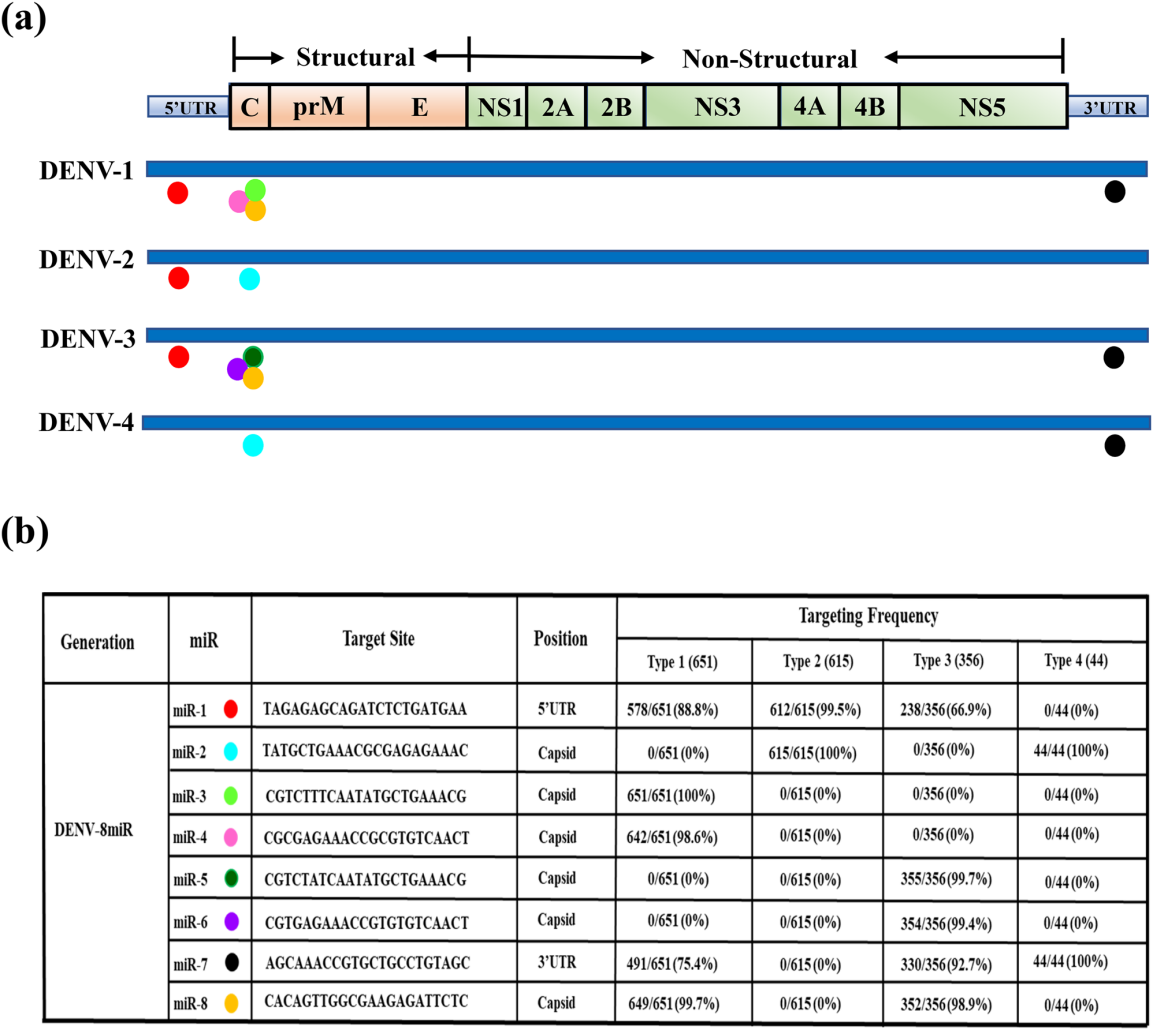

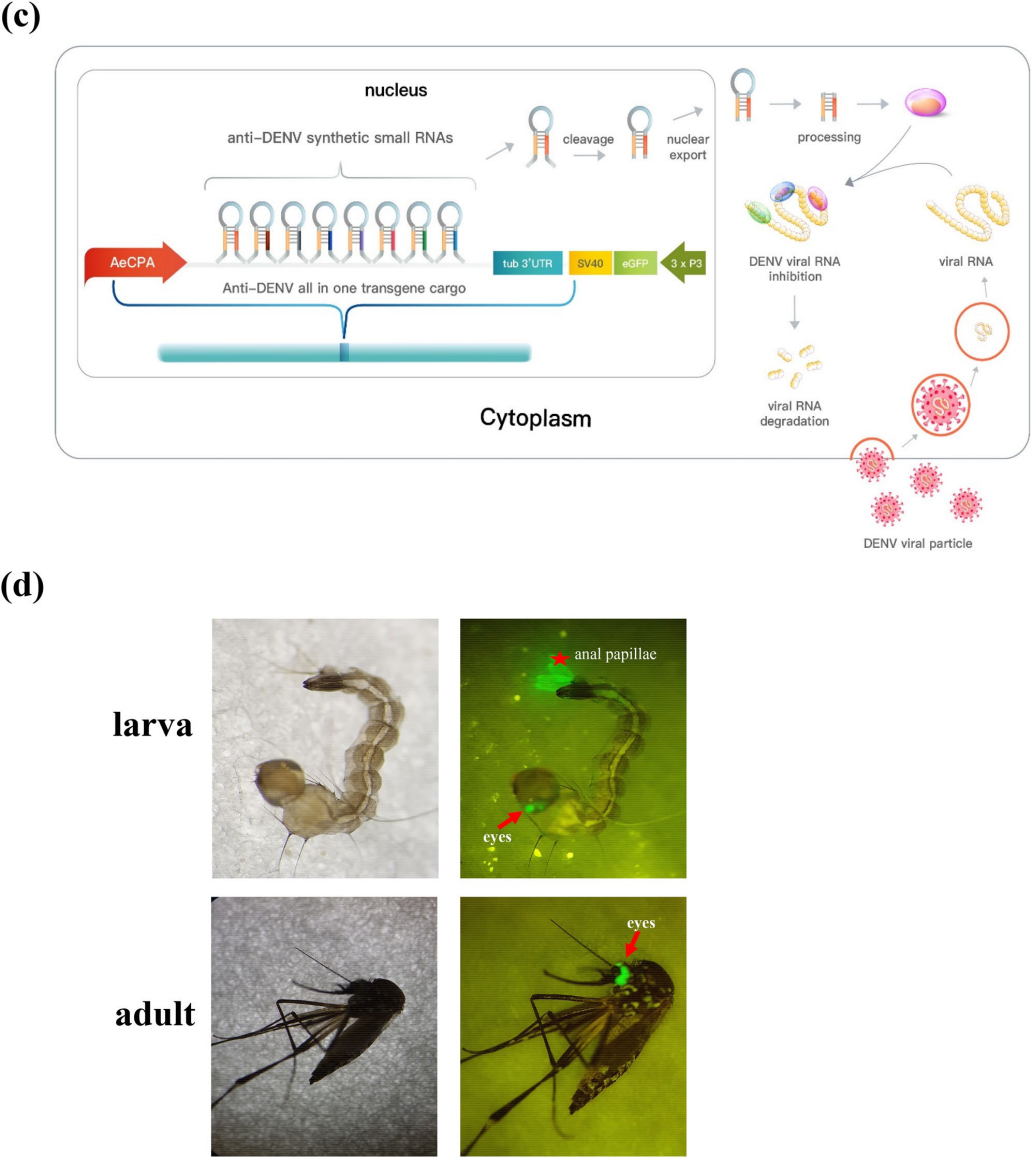
Sequence of the designed antiviral miRNAs was based on DENV consensus genomic sequence. (**a**) DENV target genes. Through the mariner (*Mos1*) transposase, ubiquitous and midgut-specific induction promoters drive the expression of a polycistronic cluster of eight synthetic small RNAs that were engineered to target conserved genes in the DENV genome. A genomic integration system was used to produce a series of transgenic mosquitoes that can express antiviral miRNAs. The DENV-8miR plasmid contains eight miRNAs: miR_8-1 targeting the 5′UTR, miR_8-7 targeting the 3′UTR, and miR_8-2 to miR_8-6 and miR_8-8 targeting the capsid, as indicated with colored circles. (**b**) The binding site coverage and similarity are presented in the table. Five types of miRNAs for DENV-1 (Myanmar strain), two types of miRNAs for DENV-2 (NGC strain), five types of miRNAs for DENV-3 (98TW503 strain), and two types of miRNAs for DENV-4 (H241 strain) were complementary targets to DENV. The viral genome sequences of each serotype were downloaded from the Viral Bioinformatic Resources Center (http://athena.bioc.uvic.ca/) and then aligned using CLC bio-Genomics software version 9.5.3 (CLC Bio, https://www.qiagenbioinformatics.com/). (**c**) Schematic of anti-DENV miRNA production. Schematic of the anti-DENV transgene, consisting of an *AeCPA*/*AePUB* promoter that drives expression of the polycistronic cluster of eight synthetic small RNAs that were engineered to target conserved genes in the DENV genome. (**d**) Screening of transgenic mosquitoes under fluorescence microscopy. The EGFP reporter driven by the 3 × P3 promoter was expressed in the eyes or anal papillae of transgenic larvae lines. Examples of EGFP reporter expression in larval and adult stages under natural (left) and blue light (right) conditions. Arrows indicate eyes and asterisks indicate anal papillae regions with EGFP expression.

To identify the most highly conserved regions of each serotype of the DENV genome for constructing the silencing cassette, we analyzed the viral genome collection of the Viral Bioinformatic Resources Center (VBRC; http://athena.bioc.uvic.ca/), and the serotype sequences were aligned and blasted separately using CLC bio-Genomics software (QIAGEN Digital Insights). The miRNA targeting sequences were designed based on DENV consensus sequences. Eight regions of DENV-1 to DENV-4 flanked by the 5′ untranslated region (5′UTR), capsid, and 3′UTR were selected as the miRNA targeting sites (Fig. 1a). The sequence coverage of the anti-DENV miRNAs relative to the viruses used to generate the consensus sequence was 66.9–100% (Fig. 1b).

All antiviral miRNA clusters were constructed in an effort to place them under the *Ae. aegypti* polyubiquitin (*AePUB*) or *Ae. aegypti* carboxypeptidase A (*AeCPA*) gene promoters to elicit either constitutive or blood-meal-inducible midgut-specific expression of the effector molecules. The polycistronic artificial miRNAs would be expressed simultaneously under the control of the same transgenic expression cassettes; therefore, multiple copies of miRNA specific to one DENV serotype or to all four serotypes could be produced in one transgenic mosquito to induce antiviral RNAi machinery to reduce DENV replication as illustrated in Fig. 1c. We used the mariner *Mos1* transposase^30^ to generate transgenic *Aedes* mosquitoes that could express anti-DENV microRNAs, and we used a genomic integration system to produce a series of stable hereditary transgenic mosquitoes containing the enhanced green fluorescent protein (EGFP) reporter gene (Supplementary Fig. 1). The EGFP reporter was driven by the 3 × P3 promoter expressed in the eyes or anal papillae of transgenic larvae lines (Fig. 1d).Several transgenic lines were selected according to miRNA expression level. One heterozygous line for each construct (*AeCPA-8miR* and *AePUB-8miR*) was retained for further experiments (Supplementary Table 1). The transgenic cassettes in mosquito chromosomes were confirmed by Southern blot analysis using restriction enzymes that have a EcoRI/HindIII cutting site within the transgene line and [P^32^]-labeled probes complementary to the EGFP reporter gene. The results indicated that the reporter gene had been integrated into the genomes of the transgenic mosquitoes (Supplementary Fig. 2) and also confirmed that the mosquitoes carried the artificial miRNA gene.

### MicroRNA expression levels in transgenic mosquitoes

**Figure 2:**
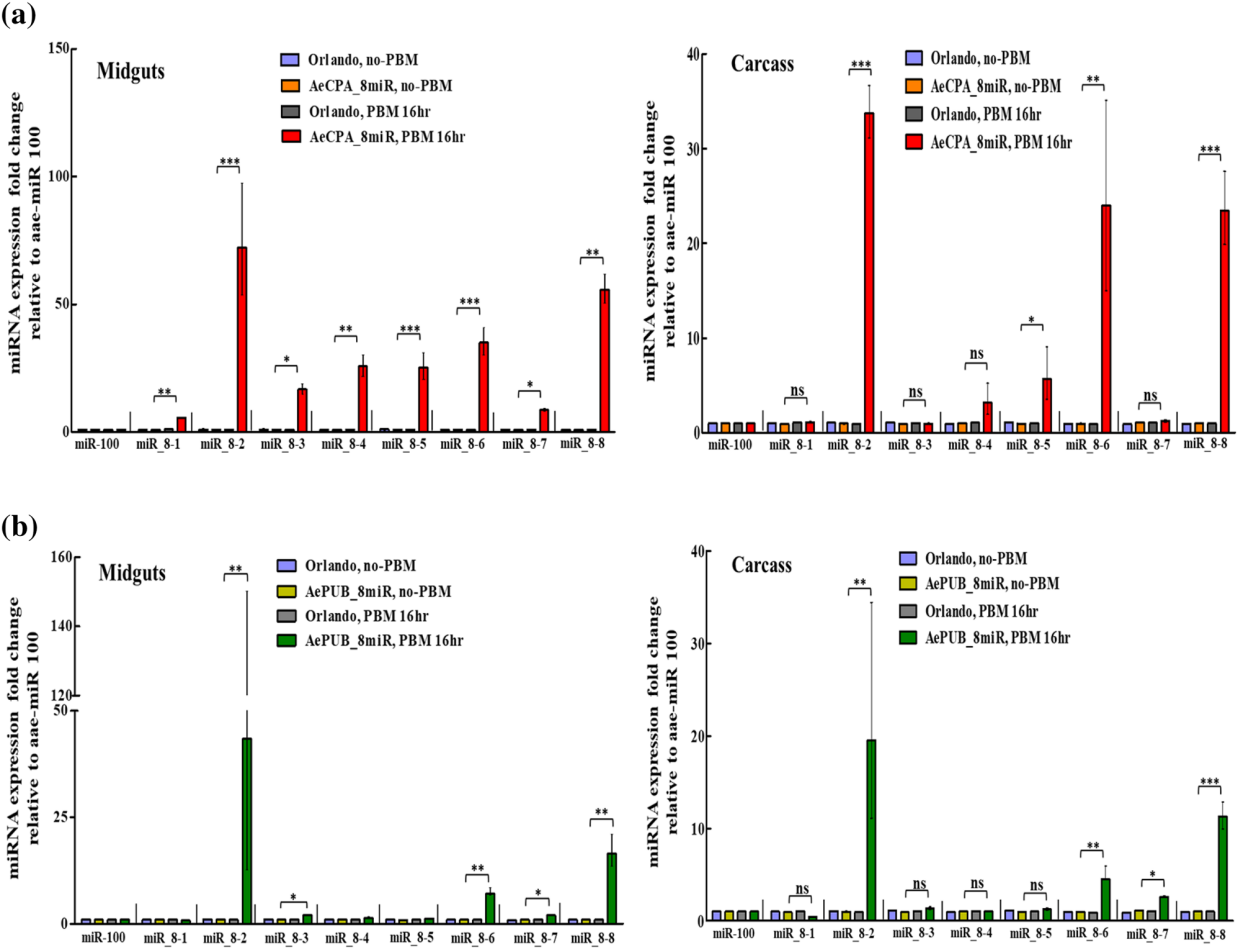
Detection of microRNA expression levels in transgenic mosquitoes. Artificial miRNAs expressed in the midgut and carcass of female transgenic mosquitoes from (**a**) *AeCPA*-8miR and (**b**) *AePUB*-8miR lines detected through qPCR. Total RNA was isolated from mosquito midguts dissected at 0 and 16 h post blood meal (PBM). RT and qPCR were conducted as described in the “Methods” section. Data are presented as relative expression levels compared with aae-miR-100. Statistical comparisons of expression levels before and after taking a blood meal for each miRNA were made using t-tests (with adjustments for multiple comparisons). Each bar represents as mean ± SEM. Significant *p* values are indicated by asterisks: *p < 0.05, **p < 0.01, ***p < 0.001.

Clustered miRNAs typically exhibit widely varying expression levels^31–33^. To investigate the expression levels of each miRNA in the cluster in each transgenic mosquito line, we evaluated the fold change in miRNA expression in the transgenic mosquitoes using quantitative polymerase chain reaction (qPCR) analyses. The miRNA expression in the midgut and carcass tissue 16 h post blood meal (PBM) was compared with miRNA expression level in the absence of a blood meal (no-PBM) in *AeCPA*-8miR transgenic lines. We found that miR_8-2 (targets DENV-2 and -4) expression had the highest fold increase, with a fold change near 70 relative to no-PBM, and the second highest expression was noted in miR_8-8 (targets DENV-1 and -3), which increased approximately 50-fold. Expression levels of miR_8-3, -4, -5, and -6 remained modest, whereas those of miR_8-1 and 8-7 were low (Fig. 2a, left panel). The antiviral miRNAs of *AeCPA*-8miR mosquitoes were detectable with similar results in the carcass, and an increased expression level was observed 16 h PBM (Fig. 2a, right panel). As the *AePUB*-8miR transgenic line miR_8-2 was by far the most highly expressed miRNA, with a fold change about 40 times relative to no-PBM. The expression levels of miR_8-6, -7and -8 were modest, whereas those of miR_8-1, -3, -4, and -5 were relatively low (Fig. 2b left panel). In the carcass tissue, the antiviral miRNAs were also detected (Fig. 2b, right panel). We interpret these data to indicate that the expression of the antiviral miRNAs in the midgut and carcass remained inducible after receiving a blood meal.

### Transgenic mosquitoes suppressed DENV titer and infection rate

**Figure 3:**
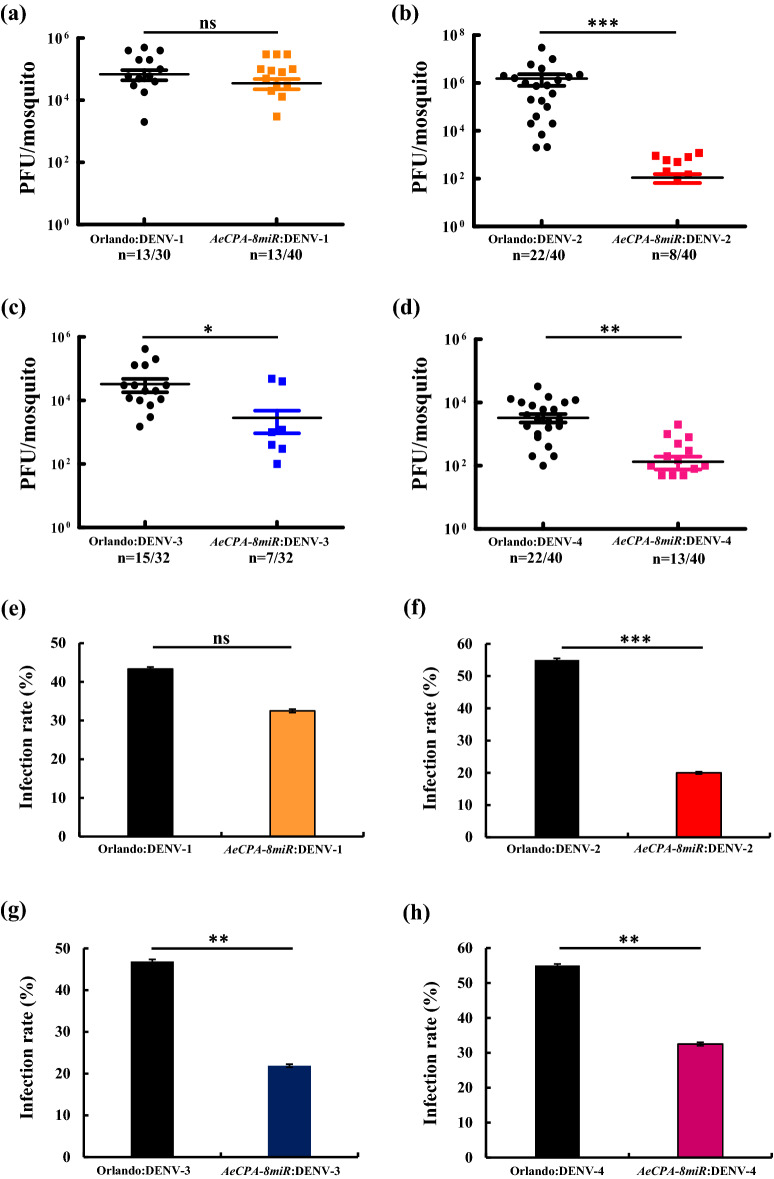
DENV challenge experiments on *AeCPA*-8miR mosquitoes at day 7. We conducted DENV challenge experiments on the transgenic *Aedes* mosquito line *AeCPA*-8miR. Seven days after a blood meal containing DENV, we collected the DENV-infected mosquitos’ midgut and performed plaque assays using BHK21 cells. We performed the virus challenges using (**a**) DENV-1 of the Myanmar strain, (**b**) DENV-2 of the NGC strain, (**c**) DENV-3 of the 98TW503 strain, and (**d**) DENV-4 of the H241 strain on the *AeCPA*-8miR and control mosquitoes (Orlando strain). For each mosquito line, the mean viral titer is plotted, and the standard error of the mean is indicated. Uninfected mosquito samples were not plotted or included when determining the mean or standard error of the mean (n = 32–40). The statistical test is the Mann–Whitney rank sum test was of GraphPad Prism (version 5.0, http://www.graphpad.com) to calculate and analyze the difference in the virus titers. (**e**–**h**) The infection rates (IRs) of the mosquito lines are indicated as percentages. IR was defined as the number of virus positive midgut samples out of the total number tested. A *t*-test was used to analyze the difference in the mosquito IRs. The error bars correspond to the confidence intervals (95%). All data are represented as mean ± SEM. Significant *p* values are indicated by asterisks: *p < 0.05, **p < 0.01, ***p < 0.001.

To test the antiviral capacity of the transgenic mosquitoes, we challenged the aforementioned two lines, *AeCPA-8miR* and *AePUB-8miR*, with all four DENV serotypes (DENV-1 of the Myanmar strain, DENV-2 of the NGC strain, DENV-3 of the 98TW503 strain, and DENV-4 of the H241 strain). We conducted the test after an incubation period of 7 or 14 days after oral feeding with a virus-infected BM. We assessed the infection rate (IR) and viral titer, and we defined IR as the number of DENV-infected mosquitoes divided by the total number of mosquitoes and measured the viral titer per mosquito. Seven days after a BM containing 10^7^ plaque-forming units (PFU)/mL of virus, the *AeCPA-8miR* line exhibited diverse antiviral capacity against the four strains (Fig. 3a–d). In the control sample (Orlando strain mosquitoes), the viral titer from the midgut of DENV-2 was approximately 1.6 × 10^6^ PFU/mL, but in *AeCPA-8miR* the average titer was approximately 10^2^ PFU/mL (Fig. 3b). For DENV-3, the average viral titer in the control sample was approximately 3.3 × 10^4^ PFU/mL, compared with approximately 2.8 × 10^3^ PFU/mL for the *AeCPA-8miR* line (Fig. 3c). Similarly, *AeCPA-8miR* was still effective against DENV-4 virus (10^2^ PFU/mL vs. 3.8 × 10^3^ PFU/mL for the control, *p* < 0.01; Fig. 3d). However, for DENV-1, no difference was noted in the viral titer between the transgenic and control mosquitoes (Fig. 3a). With respect to IR (Fig. 3e–h), the *AeCPA-8miR* line exhibited a substantially reduced IR relative to the control line for DENV-2 (20% vs. 55%), DENV-3 (22% vs. 47%), and DENV-4 (33% vs. 55%), but not for DENV-1 (33% vs. 43%). This suggests that the *AeCPA* promoter driving a cassette containing the eight anti-DENV miRNAs can suppress three of the four serotypes but not DENV-1.

**Figure 4:**
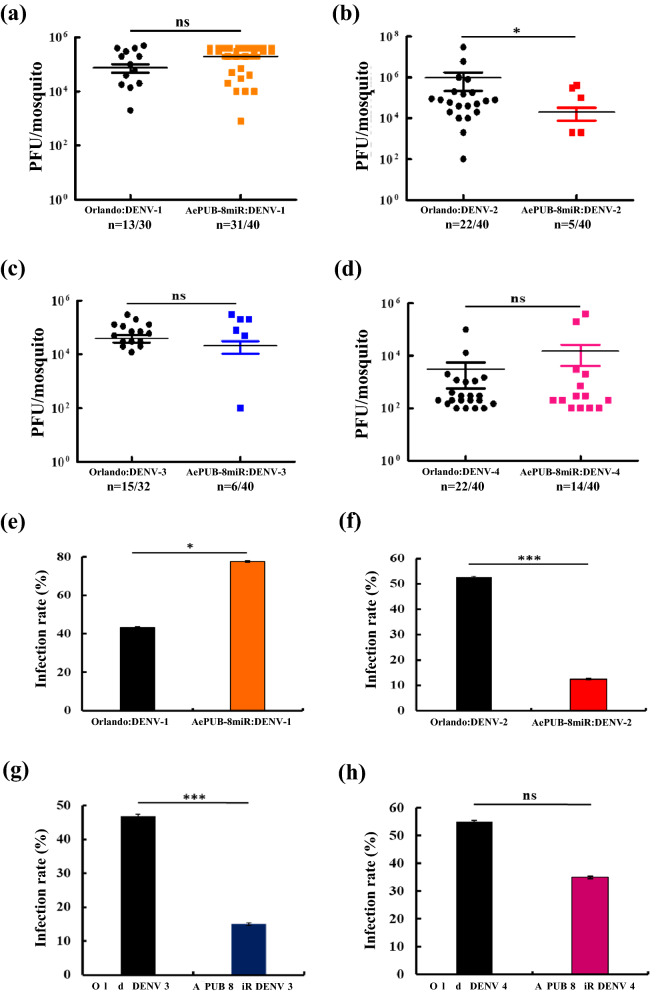
DENV challenge experiments on *AePUB*-8miR mosquitoes at day 7. We conducted DENV challenge experiments on the transgenic *Aedes* mosquito line *AePUB*-8miR. Seven days after a blood meal containing DENV, we collected the DENV-infected mosquitoes and performed plaque assays using BHK21 cells. We performed virus challenges using (**a**) DENV-1 of the Myanmar strain, (**b**) DENV-2 of the NGC strain, (**c**) DENV-3 of the 98TW503 strain, and (**d**) DENV-4 of the H241 strain on the *AePUB*-8miR and control mosquitoes (Orlando strain). For each mosquito line, the mean viral titer is plotted, and the standard error of the mean is indicated. Uninfected mosquito samples were not plotted or included when determining the mean or standard error of the mean (n = 32–40). The statistical test is the Mann–Whitney rank sum test was of GraphPad Prism (version 5.0, http://www.graphpad.com) to calculate and analyze the difference in the virus titers. (**e**–**h**) The infection rates (IRs) of the mosquito lines are indicated as percentages. IR was defined as the number of virus positive midgut samples divided by the total number tested. A *t*-test was used to analyze the difference in the mosquito IRs. The error bars correspond to the confidence intervals (95%). All data are represented as mean ± SEM. Significant *p* values are indicated by asterisks: *p < 0.05, **p < 0.01, ***p < 0.001.

In contrast to *AeCPA*, which is induced by a BM and specifically expressed in the midgut, *PUB* is expressed in all tissues. The *AePUB-8miR* strain had a lower virus titer for DENV-2 after infection (2.1 × 10^4^ PFU/mL vs. 9.7 × 10^5^ PFU/mL for the control, *p* < 0.05; Fig. 4b). However, its viral titers for DENV-1, DENV-3, and DENV-4 were similar to those of the control line (Fig. 4a,c,d). The IR for the *AePUB-8miR* line was lower than that of the control for DENV-2 and DENV-3 (13% vs. 53% and 15% vs. 47%, respectively, *p* < 0.001; Fig. 4f,g). It had a slight suppressive effect against and had an even higher IR for DENV-1 (78% vs. 43%, *p* < 0.01; Fig. 4e), whereas no effect was noted for DENV-4 (35% vs. 55%; Fig. 4h).

For the longer incubation time, 14 days post infection (dpi), our analysis revealed that the viral titers of DENV-2 and DENV-4 in the *AeCPA*-8miR line were significantly lower than in the control groups (DENV-2: 3.2 × 10^4^ PFU/mL vs. 4.6 × 10^5^ PFU/mL, *p* < 0.01; DENV-4: 1.3 × 10^3^ PFU/mL vs. 1.7 × 10^4^ PFU/mL; Supplementary Fig. 3b,d). However, for DENV-1 and DENV-3, the viral titers of the *AeCPA*-8miR line showed no differences from those of the control mosquitoes (Supplementary Fig. 3a,c). In terms of IR, *AeCPA*-8miR mosquitoes have lower IR after DENV-2 (32% vs. 65%) or DENV-4 (45% vs. 72%) infection, and higher IR after DENV-3 infection (45% vs. 97%) but no significant inhibition of DENV-1 (Supplementary Fig. 3e–h). In *AePUB*-8miR transgenic mosquitoes, the viral titer of DENV-2 was also significantly lower than in the control group (7.5 × 10^3^ vs. 9.3 × 10^5^ PFU/mL, *p* < 0.05, Supplementary Fig. 4b), but significant differences were not observed for the other three serotypes of DENV. IR analysis indicated that DENV-3 infection in *AePUB*-8miR mosquitoes was slightly lower than in the control line (25% vs. 45%, Supplementary Fig. 4g), but no effect was observed for the other three serotypes. Overall, our results indicate that the transgenic mosquitoes using either promoter had the ability to defend against DENV replication and infection.

### Repeat BM affected infection rate and viral titers

**Figure 5:**
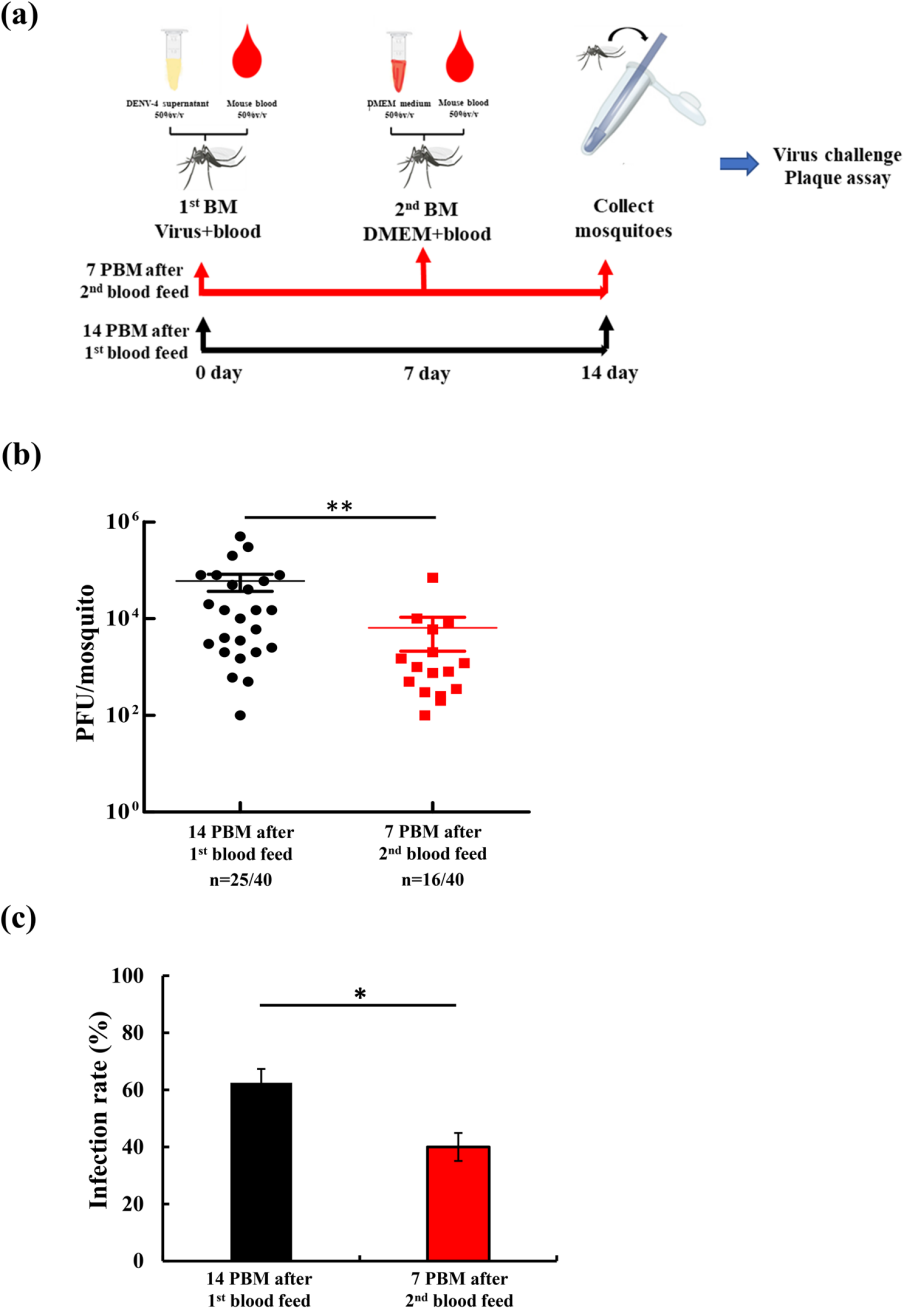
DENV challenge experiments using *AeCPA-8miR* mosquitoes administered with a second blood meal. (**a**) Schematic of the study design. A mixture containing DENV-4 supernatant and mouse blood was used to feed the *CPA* > 8miR transgenic mosquitoes at the first BM. Seven days after the blood feeding, these transgenic mosquitoes received another feeding with mouse blood not containing the virus; the DENV-infected mosquitoes were collected at day 14, and a plaque assay using BHK21 cells was performed to compare these mosquitoes with the *AeCPA-8miR* mosquitoes that received only one BM. (**b**) Viral titers of the two groups of mosquitoes. Viral titer of repeat BM versus day 14 in *AeCPA-8miR* mosquitoes. For each mosquito line, the mean number of viral titers is plotted, and the standard error of the mean is indicated. Uninfected mosquito samples were not plotted or included when determining the mean or standard error of the mean (n = 40). The statistical test is the Mann–Whitney rank sum test was of GraphPad Prism (version 5.0, http://www.graphpad.com) to calculate and analyze the difference in the virus titers. (**c**) The infection rates of the two groups of mosquitoes. Infection rate was defined as the number of positive midgut samples divided by the total number tested. The infection ratio of mosquito lines is indicated as percentages. A *t*-test was used to analyze the difference in the mosquito IRs. The error bars correspond to the confidence intervals (95%). All data are represented as mean ± SEM. Significant *p* values are indicated by asterisks: *p < 0.05, **p < 0.01, ***p < 0.001.

In the field, female mosquitoes may obtain blood meals multiple times, and this behavior increases the risk of DENV transmission and outbreaks. Since the *AeCPA* promoter is activated by blood meal uptake, we next investigated the defending efficiency of the eight miRNAs after a 2nd blood meal. We first infected mosquitoes with 10^7^ PFU/mL DENV-4 at the first BM and then incubated the successfully blood-fed mosquitoes for seven days. After incubation, mosquitoes consumed another BM which was virus-free. These two BMs activated the *AeCPA* promoter to express the 8 miRNAs to silence DENV genomes (Fig. 5a). qPCR results show that miRNAs were expressed after both BMs in midgut and carcass tissue (Supplementary Fig. 5). The antiviral capacity test revealed that, compared with the transgenic mosquitoes that did not have a second BM, the mosquitoes that received a second BM exhibited a slight but significant reduction in viral titer (5.8 × 10^4^ PFU/mL vs. 7.1 × 10^3^ PFU/mL, *p* < 0.01; Fig. 5b) and a 22% lower IR (from 62.5 to 40.0%, *p* < 0.05; Fig. 5c). These results indicate that the second blood meal potentially increases the blocking efficiency of DENV in refractory transgenic mosquitoes.

### Virus transmission efficiency in transgenic mosquitoes

**Figure 6:**
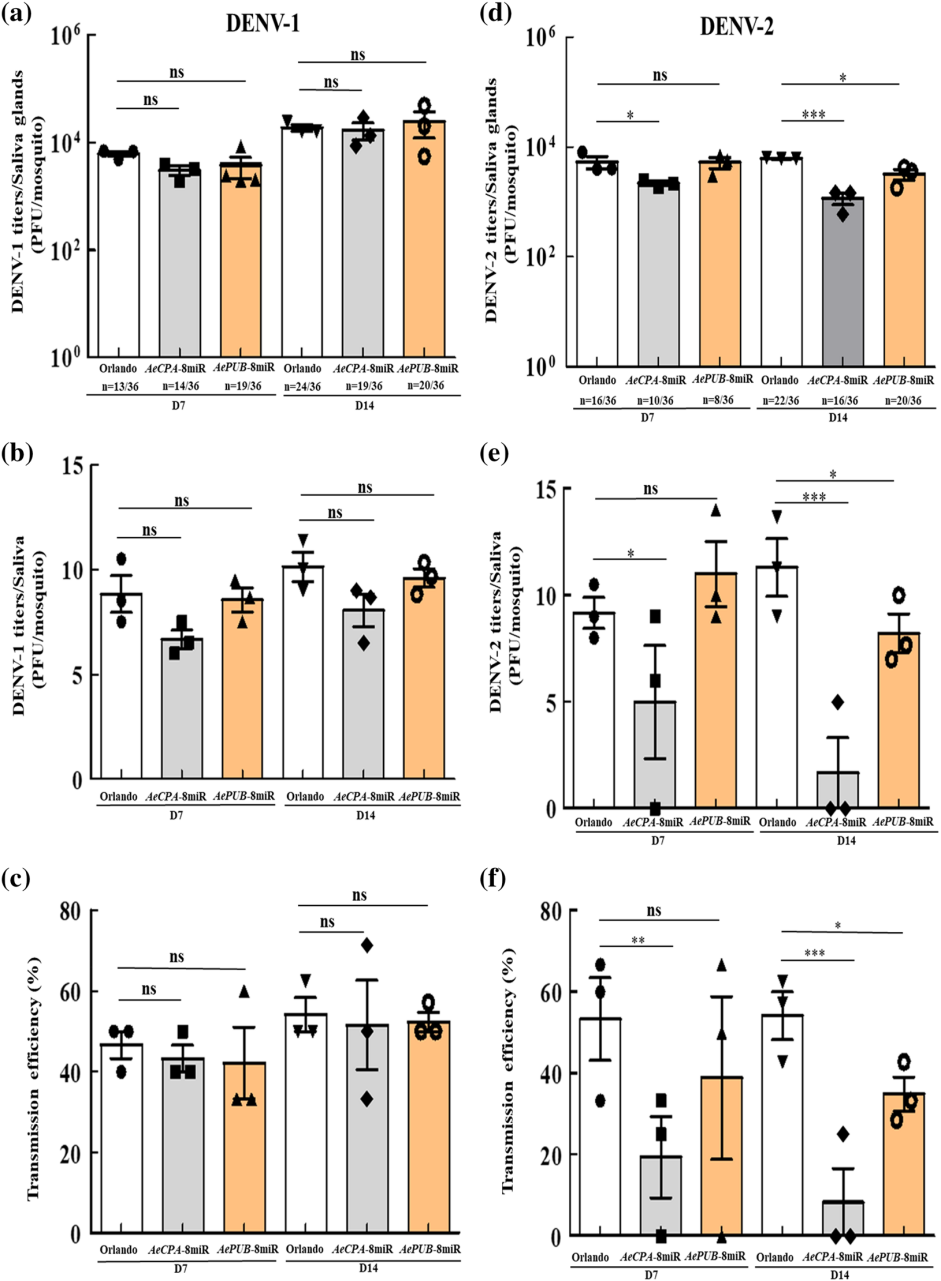

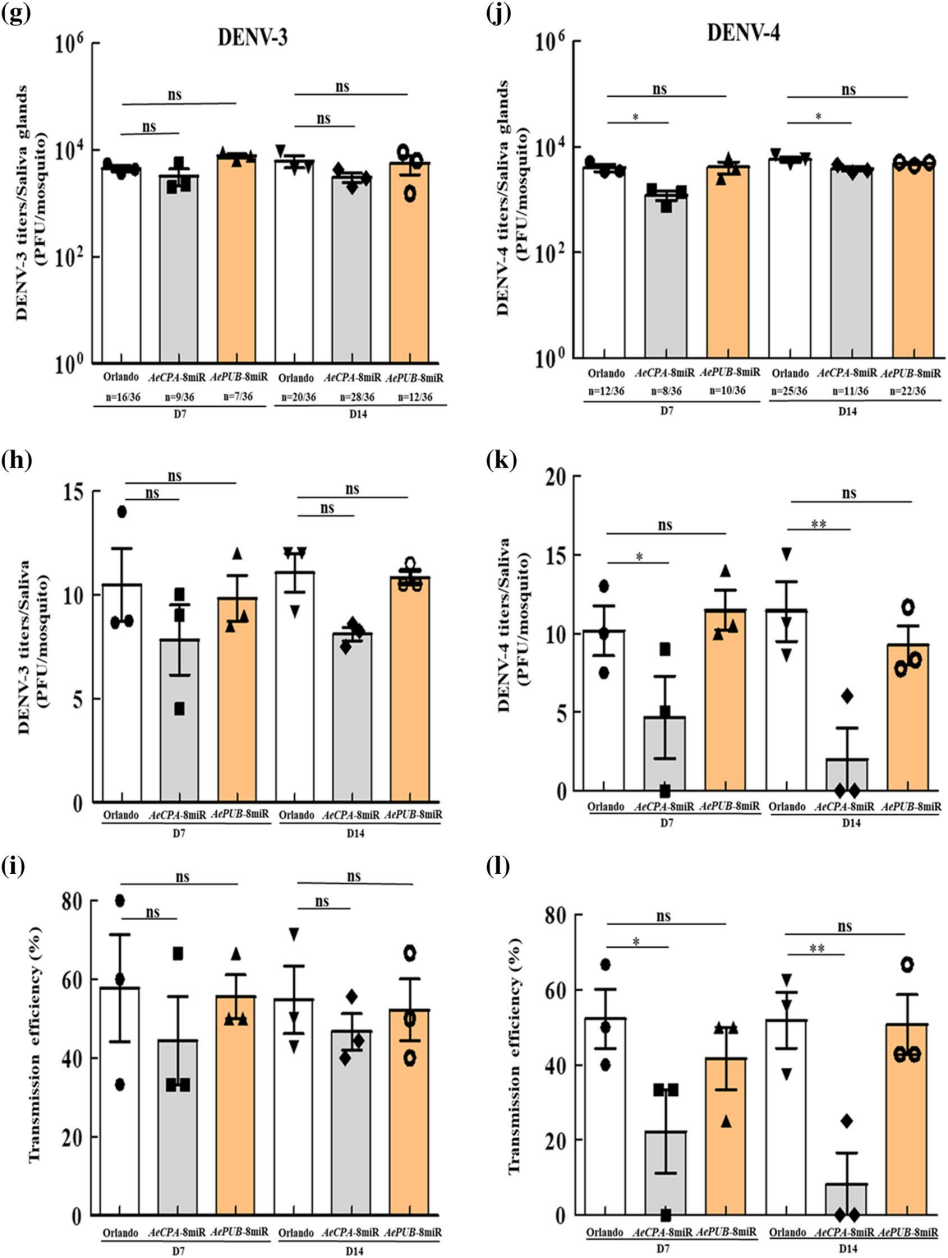
Transgenic mosquitoes have reduced virus transmission potential. At 7- and 14-dpi with DENV-1 to -4, the salivary glands and the saliva were collected in female transgenic and control (Orlando strain) mosquitoes. Viral suppression was determined by plaque assay. (**a**,**d**,**g**,**j**) In salivary glands. (**b**,**e**,**h**,**k**) In saliva. Saliva samples were collected after 30 min in a P20 tip containing 5 μL of FBS and then expelled into 45 μL of L-15 media for analysis. For each mosquito line, the mean viral titer is plotted, and the standard error of the mean is indicated. Uninfected mosquito samples were not plotted or included when determining the mean or standard error of the mean. Each sample corresponds to three replicates (3 × 12 mosquitoes). The statistical test is the Mann–Whitney rank sum test was of GraphPad Prism (version 5.0, http://www.graphpad.com) to calculate and analyze the difference in the virus titers. (**c**,**f**,**i**,**l**) The transmission efficiencies of infection of the mosquito lines are indicated as percentages, which are defined as the number of positive saliva samples (i.e., successful transmission events) divided by the number of tested samples. A t-test was used to analyze potential differences in the mosquito transmission efficiency assay. The error bars correspond to the 95% confidence intervals. All data are represented as mean ± SEM. Significant *p* values are indicated by asterisks: *p < 0.05, **p < 0.01, ***p < 0.001.

To evaluate the transmission ability of these transgenic lines, the saliva of orally infected mosquitoes was examined. Mosquitoes were infected orally with DENV-1 to -4 and then incubated for 7 or 14 days, after which the salivary glands and saliva of these mosquitoes were collected to test the virus titers and transmission efficiency (Fig. 6). In the salivary glands of the *AeCPA*-8miR line, DENV-2 and DENV-4 viral titers were significantly lower both 7 and 14 dpi when compared to controls (Fig. 6d,j). However, for DENV-1 and DENV-3, no difference was noted in the viral titer between the transgenic and control mosquitoes (Fig. 6a,g). For the *AePUB*-8miR line, we found reductions in only DENV-2 at 14 dpi (Fig. 6d), but showed no significant changes for any of the other three serotypes of DENV. After testing for potential suppressive effects in the salivary glands, we next tested viral titer in saliva. In the saliva, we found that *AeCPA*-8miR showed significantly reduced viral titers for DENV-2 and DENV-4 at 7 and 14 dpi (Fig. 6e,k). On the other hand, *AePUB*-8miR was shown to reduce the virus titer of DENV-2 at 14 dpi, but there was no significant difference in virus titer at 7 dpi (Fig. 6e). For DENV-1 and DENV-3, neither *AeCPA*-8miR and *AePUB*-8miR line showed a significant suppressive effect (Fig. 6b,h). At 7 dpi, the DENV-2 transmission efficiency was, on average, 19.4% ± 14.1% and 38.9% ± 22.3% in the *AeCPA*-8miR and *AePUB*-8miR lines, respectively, and the control (Orlando) mosquitoes had an average transmission efficiency of 53.3% ± 14.4% (Fig. 6f). At 14 dpi, we observed that the transgenic lines had a significantly lower transmission efficiency than the control mosquitoes (Orlando: 54.2% ± 8.3%, *AeCPA*-8miR: 8.3% ± 11.8%, *AePUB*-8miR: 34.9% ± 5.9%) (Fig. 6f). We also found similar results in the transmission efficiency for the *AeCPA*-8miR line was lower than that of the control for DENV-4 at 7 and 14 dpi (22.2% ± 15.4% vs. 52.2% ± 11.9% and 8.3% ± 11.8% vs. 51.9% ± 11.5%, respectively, p < 0.01; Fig. 6l). However, for DENV-1 and DENV-3, the transmission efficiency of the *AeCPA*-8miR and *AePUB*-8miR line showed no differences from those of the control mosquitoes (Fig. 6c,i). In these transgenic lines, the transmission efficiency of DENV-2 and DENV-4 is significantly affected by *AeCPA*-8 miRNA, while that of DENV-1 and DENV-3 is not affected.

## Discussion

Dengue fever is one of the most common and widespread mosquito-borne diseases in the world. It contains four antigenically distinct serotypes (DENV-1 to -4), any one of which could be the major serotype in an epidemic. Different approaches for preventing mosquitoes from being infected with DENV had been demonstrate^34–36^. However, each approach only targeted a specific serotype, which makes these previous methods insufficient and impractical. In this study, we generated two transgenic mosquito lines that expressed antiviral microRNAs, which were designed to target the genomes of the four DENV serotypes under the control of the *Ae. aegypti* PUB and CPA promoters. After viral challenge, the viral titer and IR suggested a significant suppression of three serotypes of DENV infection in *AeCPA*-8miRNA transgenic lines and one serotype of DENV infection in *AePUB*-8miRNA transgenic lines at day 7 PBM compared to wild-type (Orlando strain) mosquitoes. After a second blood meal, the *AeCPA*-8miRNA line retained its effective antiviral activity at day 14 post infection. The virus transmission efficiencies in the *AeCPA*-8miRNA line were significantly reduced compared with both the *AePUB*-8miRNA and the control mosquitoes at 14 days PBM. In summary, our study reveals that miRNA-based genetically engineered mosquitoes had reduced viral titers, IRs, and transmission efficiency for the most of the four DENV serotypes.

Both of the transgenic mosquito lines that were generated here had one copy of the DENV miRNA cassette (heterogeneous, data not shown). Our results demonstrate that the heterogeneous DENV-8 miRNA transgenic mosquito lines were able to obtain refractoriness to DENV infection and transmission, with the result that the infection rates of some of the four serotypes (types 2, 3, and 4 in *AeCPA*-8miR; 2 and 3 in *AePUB*-8miR; Figs. 3 and 4) of DENV were significantly lower than the infection rates of the corresponding serotype in the Orlando strain. Furthermore, we found that the DENV-4 infection in the salivary gland of the *AeCPA*-8miR line was significantly lower than in the Orlando strain, resulting in the viral load in the saliva being reduced by 93% (from 36.1 to 2.7%) when compared to Orlando mosquitoes. Similar results have also been found in previous research^32^, which demonstrated that a heterogeneous line of miRNA transgenic mosquitoes were able to reduce ZIKV infection and transmission. Lower infection and transmission rates in mosquitoes represent a lower risk of virus dissemination, and this can be achieved in a heterogeneous background. We believe modifying the miRNA sequences or promoter strength may provide a higher suppression ability against DENV infection and transmission.

After mating, female mosquitoes are able to ingest multiple blood meals^37,38^. Once infected by DENV, and after the virus has entered the salivary glands, females are able to transmit DENV during subsequent biting events for the rest of their lives^39^.The CPA promoter is not a continuous expression promoter; its activity is triggered by a blood meal. From the single oral DENV infection/blood meal results (Fig. 3), we found that viral load and infection rate of DENV in the CPA promoter transgenic line were significantly lower than those of Orlando mosquitoes. In the *AeCPA*-8miR line, after the blood meal is digested, antiviral protection is likely to be lost because the miRNA expression will cease. Virus infection in mosquito body will resume a natural state. We then stimulated the expression of the miRNAs again (Supplementary Fig. 5) by feeding another blood meal to these mosquitoes seven days after first blood meal. In these mosquitoes, we found that the viral load and infection rate were significantly lower than in those that were only fed a blood meal once (Fig. 5). These results demonstrated that whether these miRNAs were expressed during or after DENV infection, they were capable of suppressing DENV replication.

In our result, the expression level of each miRNA of DENV-8miR cluster were different (Fig. 2). In a cell, several miRNAs existed as a cluster was not hard to see, each cluster was under controlled by a promoter, however, the expression levels of these miRNAs are different^31,40–42^, even though they are all produced from the same transcript and driven by one promoter. There are some possible reasons to make this variation of expression, the architecture of miRNAs, including the dimensional structure of the hairpin, the spacing sequence and the length of spacing are all crucial importance for the knockdown efficiency. In the miRNA processing, the guide strand is the one that is used in the RISC for RNA interference^43,44^. However, how the RISC chooses the guide strand (the functional strand for silencing) from the individual miRNA precursor was dependent on their individual sequence. Based on these results, although the knockdown efficacy in this study did not achieve to the same level for all four different genomes of DENV. In the future, we may optimize biophysical properties and/or sequence of the spacer, spacer, and select the more efficient target sequence to optimize knockdown efficiency for all DENV genome.

The results of virus challenge and plaque assay experiments revealed that DENV infection was reduced to around 50% in the transgenic mosquitoes. Both of the transgenic lines showed effective antiviral activity against DENV-2 and DENV-4 infection either at 7- or 14-days post infection, but not to DENV-1 infection (Figs. 3 and 4 and Supplementary Figs. S3 and S4). This may be due to the high expression level of miR_8-2 in both transgenic lines (Fig. 2). The sequence of miR_8-2 is designed to target genomic sequences present in both DENV-2 and DENV-4, resulting in the effective suppression of DENV replication. From the viral load results and DENV-1 infection rates, we can conclude that the function of miR_8-3, miR_8-4, and miR_8-8 may not be sufficient to provide antiviral activity, even though the expression level of miR_8-8 was the second highest among the eight miRNAs. miR_8-8 targets both DENV-1 and DENV-3, however DENV-3 is also targeted by two additional miRNAs, miR_8-5 and miR_8-6, which do not have targets within the DENV-1 sequence. Antiviral activity against DENV-3 in the transgenic lines may have contributions from these two miRNAs. miR_8-1 and miR_8-7 were designed to target multiple serotypes of DENV, including DENV-1, however, their expression was the lowest of these eight miRNAs, which may have restricted their efficiency in anti-DENV activity. The actual conditions of how these eight miRNAs function in the suppression of viral load and infection rate in DENV infection are far more complicated and may include unexpected interactions, for example, mutations generated in the DENV genome sequence, the targeting positions of these eight miRNAs within the DENV genome, interactions between exogenous and endogenous miRNAs, secondary structures or protein-RNA interactions, or even transcription or translation regulation^45^ by these eight miRNAs. Therefore, to improve the efficiency of suppression of DENV infection in transgenic mosquitoes, future experiments should focus on optimizing the sequences of anti-DENV miRNAs, as well as their expression and processing.

Another factor that affects DENV replication is temperature, which may also affect the efficacy of RNA silencing^46,47^. Previous studies have revealed that the extrinsic DENV-2 incubation period in *Ae. aegypti*, *Ae. albopictus*, and *Culex pipiens* was reduced at higher temperatures^48–50^. In other words, a short extrinsic incubation period may increase the capacity of a vector to transmit the virus. In the present context of global warming, the DENV may have a shorter extrinsic incubation period in mosquitoes. In future studies, virus challenge experiments should therefore be performed at higher temperatures to elucidate the antiviral capacity of transgenic mosquitoes more comprehensively.

This study demonstrated that the expression of DENV sequence specific miRNAs in transgenic *Ae. aegypti* decreased DENV infectivity. These eight miRNAs were designed to target genome sequences in all four serotypes of DENV separately. Their expression improved the RNAi pathway’s effectiveness in the fight against DENV and resulted in an effectively reduced viral load and infection rate in transgenic mosquitoes. The transmission efficiency of DENV in transgenic mosquitoes was decreased as well. This strategy may be useful for combating the spread of DENV or other arboviruses.

## Methods

### Mosquito rearing

*Aedes aegypti* (Orlando strain, which was from the University of Maryland Insect Transformation Facility) were reared in an insectary under the following conditions: 28 ℃, 70%–80% relative humidity, and a 12-h dark–light regime. Approximately 100 adults were pooled and maintained in a 30 × 30 × 30 cm acrylic cage with 10% sucrose as a source of dietary sugar. Adult mosquitoes shifted to feeding on blood from a mouse donor (BALB/c; BioLASCO Taiwan Co., Ltd., Taipei, Taiwan). Three days after the BM, the mosquitoes were allowed to lay eggs on wet filter paper, which was then dried and stored.

### miRNA-based RNAi design

In order to design artificial miRNAs targeting the viral genomic RNA of the four serotypes (1, 2, 3, and 4) of DENV, the viral genome sequences of each serotype were downloaded from the Viral Bioinformatic Resources Center (http://athena.bioc.uvic.ca/) and then aligned using CLC bio-Genomics software version 9.5.3 (CLC Bio, QIAGEN, https://www.qiagenbioinformatics.com/). The downloaded viral genome sequences included 651 sequences of serotype 1, 615 sequences of serotype 2, 356 sequences of serotype 3, and 44 sequences of serotype 4. Each serotype was aligned separately. The alignment results revealed several highly conserved regions located within the 5′UTR, capsid, or 3′UTR in the viral genomes. We selected miRNA sequences from these highly conserved regions based on their GC content and the length of the conserved region. These miRNA sequences were then verified in their sequence identity to each serotype via alignment with genome sequences using UltraEdit (IDM Computer Solutions). The chosen miRNA sequences were then cloned into the miR6.1 backbone, which was derived from *Drosophila melanogaster*^51^, to generate the miR-based RNAi cassette for silencing DENV in mosquitoes. The sequences of these eight miRNAs are listed in Supplementary Table 2.

### Plasmid construction

We first generated tandem miRNA coding DNA fragments. Double-stranded DNA fragments of eight DENV miRNAs were composed using paired oligonucleotides (Supplementary Table 3) through annealing and extension, followed by another PCR amplification with a primer set: miR6.1_5′ EcoRI/BglII and miR6.1_3′ XhoI/BamHI, to produce miRNA units that contained restriction enzyme cutting sites. These eight miRNA units were assembled to become a tandem miRNA coding DNA fragment using BglII and BamHI sites and then subcloned into the EcoRI/XhoI sites of pMOS1_*AePUb*-CLEC18A-2xHA_3xP3-eGFP^40^ to create pMOS1_nanos-DENV-8miR_3xP3-EGFP constructs. To finish the construction, promoter sequences of *AeCPA* and *AePUB* were amplified from the genomic DNA of *Ae. aegypti* using a CPA primer set (*AeCPA*-F and *AeCPA*-R) and a PUB primer set (*AePUB*-F and *AePUB*-R). The amplified DNA products were then cloned into the FseI and EcoRI sites of pMOS1_nanos-DENV-8miR_3xP3-EGFP constructs with In-Fusion HD Cloning technology (Clontech), resulting in pMOS1-*AeCPA*-DENV-8miR-3xP3-EGFP and pMOS1-*AePUB*-DENV-8miR-3xP3-EGFP constructs. Both plasmids were then used as donor plasmids for embryo microinjection.

### Generation of transgenic mosquitoes

To establish the *AeCPA*-8miR and *AePub*-8miR transgenic mosquito lines, donor and helper plasmids were mixed with injection buffer (5 mM KCl and 0.1 mM NaH_2_PO_4_, pH 6.8) at concentrations of 1000 ng/μL and 600 ng/μL, respectively, and then microinjected into preblastoderm-stage embryos, as described in a related study^52^. Injected embryos were then hatched and reared under standard conditions. All the generation 0 (G0) eggs were collected. We used a fluorescent microscope (SZX12; Olympus) to screen G0 mosquito larvae to identify those with the EGFP fluorescent marker in their eyes and anal papillae, a pattern driven by the 3xP3 promoter. Positive G0 males were outcrossed to wild type females (Orlando strain) in a ratio of 1:3. G1 mosquitoes positive for the reporter were self-crossed by placing a single male with a single female. Eggs from G1 were collected, and the genotypes of the G1 parents were ascertained to distinguish the progeny genotypes. The progeny larvae of the following two generations were screened for eye-specific EGFP reporter gene expression to confirm their genotype. Further, we confirmed the integration of the transgenic cassettes into mosquito chromosomes by sequencing and Southern blot and used the heterozygous transgenic lines (expressing the eight miRNAs) as the experimental groups in the subsequent studies.

### Southern blot analysis

The genomic DNA extractions were conducted in accordance with the manufacturer’s protocol (Invitrogen). Approximately 2.5 µg of extracted total genomic DNA was digested by EcoRI/HindIII, followed by DNA separation on 0.8% agarose gel. The separated DNA was then transferred onto a nylon membranes and hybridized overnight at 42 ℃ with the oligonucleotide’s probes, whose cover miR-1 to miR-8 coding sequences, respectively (Supplementary Table 4). We mixed theses eight DNA oligonucleotide with equal molars and performed 5′ ^32^P-labeling by T4 polynucleotide kinase for hybridization. The expected size of hybridization pattern was 1970 bp digested in *AeCPA*-8miR and *AePUB*-8miR mosquitoes, respectively.

### DENV

DENV serotypes 1–4 were propagated in C6/36 (from *Ae. albopictu*s) cells. Briefly, C6/36 cells, grown in a 175 mm^2^ flask at a confluency of approximately 80% at 28 °C, were infected at a multiplicity of infection of 0.5 for 3 h at 28 °C. Later, fresh medium was added and the infection proceeded for 5 days at 28 °C. Finally, the cell culture supernatant was filtered through a 0.22-μm filter, aliquoted, and stored at − 80 °C until use.

### Oral DENV infection of mosquitoes and 2nd blood feeding

For DENV oral infection, virus supernatant from each serotype of DENV-infected C6/36 cells was mixed 1:1 with mouse blood to yield 10^7^ PFU/mL virus blood. This virus-containing blood was then provided to 150 female mosquitoes (Orlando, *AeCPA*-8miR, or *AePUB*-8miR lines at 7 to 10 days post-emergence) by artificial blood feeding for 40 min. Successfully blood-fed mosquitoes, which can be identified easily by visual cues, were separated from non-blood-fed mosquitoes and then incubated for 7 or 14 days at 28 °C and 80% relative humidity.

For the 2nd blood feeding experiment, Orlando or *AeCPA*-8miR mosquitoes at seven days post DENV infection were provided with another blood meal lacking the virus supernatant through artificial blood feeding for 40 min. Mosquitoes that were successfully blood-fed were transferred to a new cage and then incubated for the designated days.

### Plaque assay

Viral plaque assays for both virus stocks and infected mosquitoes were performed using previously published protocols^53^. BHK-21 cells were seeded into six-well plates at 2.5 × 10^5^ cells per well, with the addition of 2 mL of 5% fetal bovine serum (FBS) in RPMI medium. Plates were incubated at 37 °C for 16–24 h before the medium was removed, and 100 µL of a tenfold serial dilution of the virus was layered on top of the cells. Plates were shaken gently and incubated at 37 °C for 2 h. After absorption, we removed the infectious medium, and 3 mL of 1% methylcellulose (MC) medium (with 2% FBS, 100 mM sodium pyruvate, 1 M HEPES in RPMI medium, pH 7.4) was added to each well. Samples were incubated at 37 °C with 5% CO_2_ supplemented for 5 days. Plates were harvested when plaques were visible to the naked eye. When the incubation was completed, the MC medium was removed and the plates were stained through the addition of 1% crystal violet solution to each well. Staining was performed for at least 3 h. Plates were subsequently rinsed and plaques were counted.

### DENV transmission assay

Female mosquitoes of each experimental groups were infected orally with 10^7^ PFU/mL of DENV and then incubated for 7 or 14 days. Saliva of these infected mosquitoes were collected accordingly^31,54^. Briefly, legs and wings of the infected mosquito was removed, followed by inserted its mouthpiece into a P200 tip which was filled with 5 µL of FBS. 30 min later, FBS in the tip was added into 45 µL of serum-free medium in RPMI and then analyzed with plaque assay. Tissues of this mosquitoes were subjected to the plaque assay as well. Transmission efficiency was calculated as follow:

$$\text{Transmission efficiency}=\frac{\text{Number of mosquitoes with infectious saliva}}{\text{Number of examined mosquito}}$$

### qPCR

Mosquito small RNAs were extracted using Tri-reagent (Merck) following the manufacturer’s protocol. The reverse transcription (RT) of extracted RNAs (20 µL) proceeded as follows: the extracted RNA was mixed with the stem-loop RT primer (Supplementary Table 5), and the dNTPs were denatured at 65 °C for 5 min and then immediately cooled on ice. The treated RNA mixtures were incubated at 16 °C for 30 min followed by pulsed RT (30 °C for 30 s, 42 °C for 30 s, and 50 °C for 1 s for 60 cycles; terminated by 5 min at 85 °C) to increase detection sensitivity. The miRNA analysis used 2 µL of cDNA as a sample. Analysis was performed using a Universal Probe Library probe assay (Universal Probe Library probe #21) with qPCR (ABI PRISM 7900HT Sequence Detection System). Amplification proceeded with an initial step at 50 °C for 2 min; a denaturing step at 95 °C for 10 min; and 40 cycles of amplification at 95 °C for 15 s and 60 °C for 1 min. qPCR data was normalized against aae-U6 (as the reference gene) and used the endogenous miRNA aae-miR-100 as an endogenous control to obtain the expression profiles of anti-DENV miRNAs. Primers that were used in the miRNA analysis are listed in Supplementary Table 6.

### Statistical analysis

Statistical Package for GraphPad Prism version 5.0 (http://www.graphpad.com) and Microsoft Excel were used for data entry, processing, and statistical analysis. Analysis of variance with a post-hoc Tukey’s test was used to compare differences in means between different treatments. The Mann–Whitney rank sum test was used to analyze the difference in the titer of each virus between the wild-type (Orlando strain) and transgenic (*AeCPA-8miR* and *AePUB-8miR*) mosquitoes. The t test was used to analyze the difference in mosquito IR. A *p* value < 0.05 was considered significant.

## Supplementary Information

Supplementary Information.

## Data Availability

All data needed to evaluate the conclusions in the paper are present of the paper and/or the Supplementary Materials. Additional data related to this paper may be requested from the authors.
