# Author Correction: Transgenic refractory *Aedes aegypti* lines are resistant to multiple serotypes of dengue virus

**DOI:** 10.1038/s41598-022-05054-9

**Published:** 2022-01-10

**Authors:** Wei-Liang Liu, Chia-Wei Hsu, Shih-Peng Chan, Pei-Shi Yen, Matthew P. Su, Jian-Chiuan Li, Hsing-Han Li, Lie Cheng, Cheng-Kang Tang, Shih-Hsun Ko, Huai-Kuang Tsai, Zing Tsung-Yeh Tsai, Omar S. Akbari, Anna-Bella Failloux, Chun-Hong Chen

**Affiliations:** 1 National Mosquito-Borne Diseases Control Research Center, National Health Research Institutes, Miaoli, Taiwan; 2 Institutes of Molecular and Cellular Biology, National Taiwan University, Taipei, Taiwan; 3 Graduate Institute of Microbiology, College of Medicine, National Taiwan University, Taipei, Taiwan; 4 Unit of Arboviruses and Insect Vectors, Department of Virology, Institute Pasteur, Paris, France; 5 Department of Biological Science, Nagoya University, Nagoya, Japan; 6 Institute for Advanced Research, Nagoya University, Nagoya, Japan; 7 National Institute of Infectious Diseases and Vaccinology, National Health Research Institutes, Miaoli, Taiwan; 8 Institute of Information Science, Academia Sinica, Taipei, Taiwan; 9 Section of Cell and Developmental Biology, Division of Biological Sciences, University of California, San Diego, La Jolla, CA 92093 USA; 10 National Institute of Infectious Diseases and Vaccinology, National Mosquito-Borne Diseases Control Research Center, National Health Research Institutes, Zhunan, Taiwan

Correction to:* Scientific Reports* https://doi.org/10.1038/s41598-021-03229-4, published online 13 December 2021

The original version of this Article contained an error in the spelling of the author Anna-Bella Failloux which was incorrectly given as Anna-Bella Failoux.

The original Article has been corrected.

