# Supplementary material for: Transgenic refractory Aedes aegypti lines are resistant to multiple serotypes of dengue virus
Source: Sci Rep. 2021 Dec 13;11:23865. doi: 10.1038/s41598-021-03229-4 (PMC8668939; doi:10.1038/s41598-021-03229-4)
Supplement: Supplementary file 1 — Supplementary Information. [file 41598_2021_3229_MOESM1_ESM.pptx]

## Slide 1
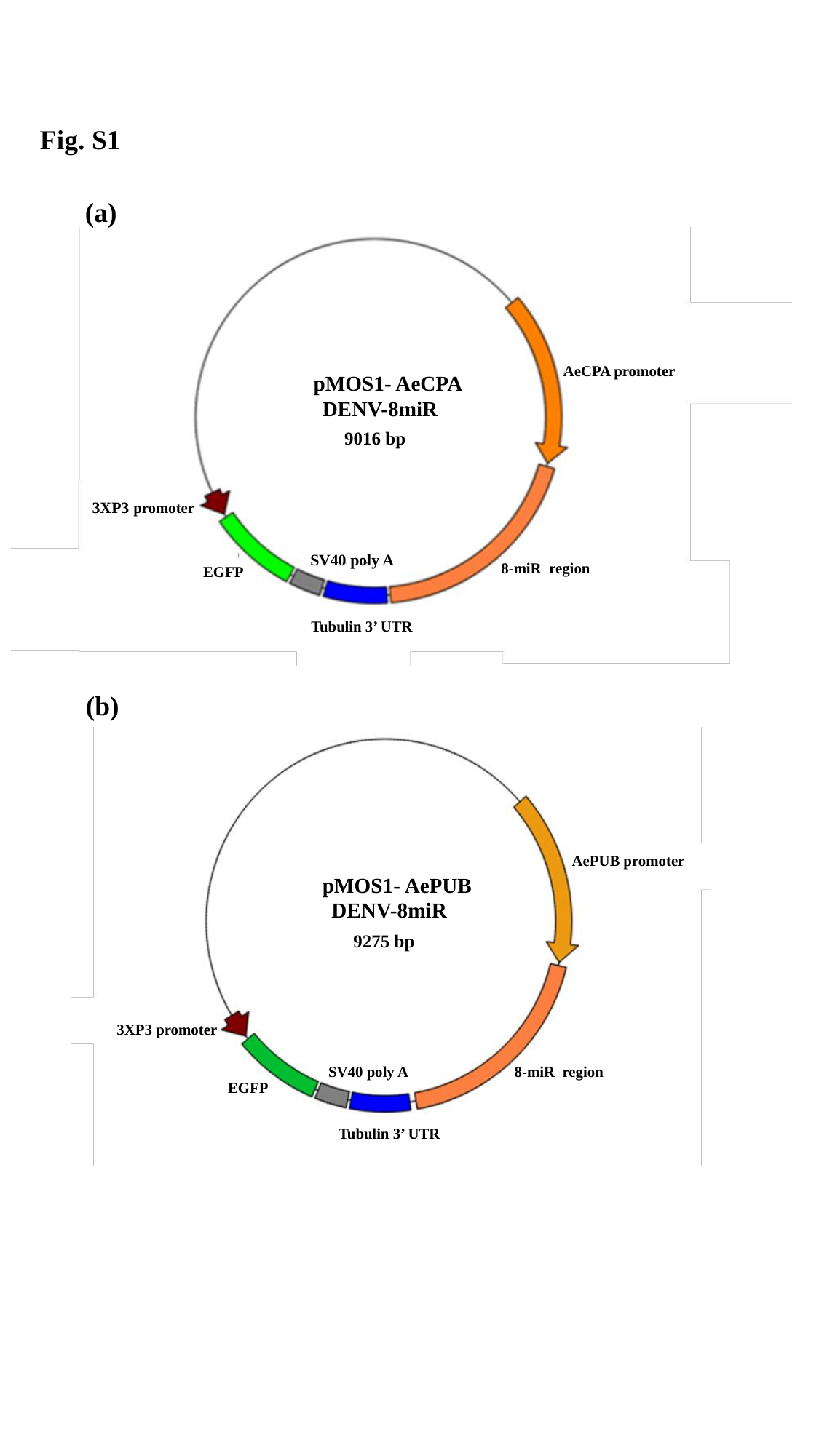

Fig. S1
(a)
AeCPA promoter
pMOS1- AeCPA
DENV-8miR
9016 bp
3XP3 promoter
SV40 poly A
8-miR region
EGFP
Tubulin 3’ UTR
(b)
AePUB promoter
pMOS1- AePUB
DENV-8miR
9275 bp
3XP3 promoter
SV40 poly A
8-miR region
EGFP
Tubulin 3’ UTR

## Slide 2
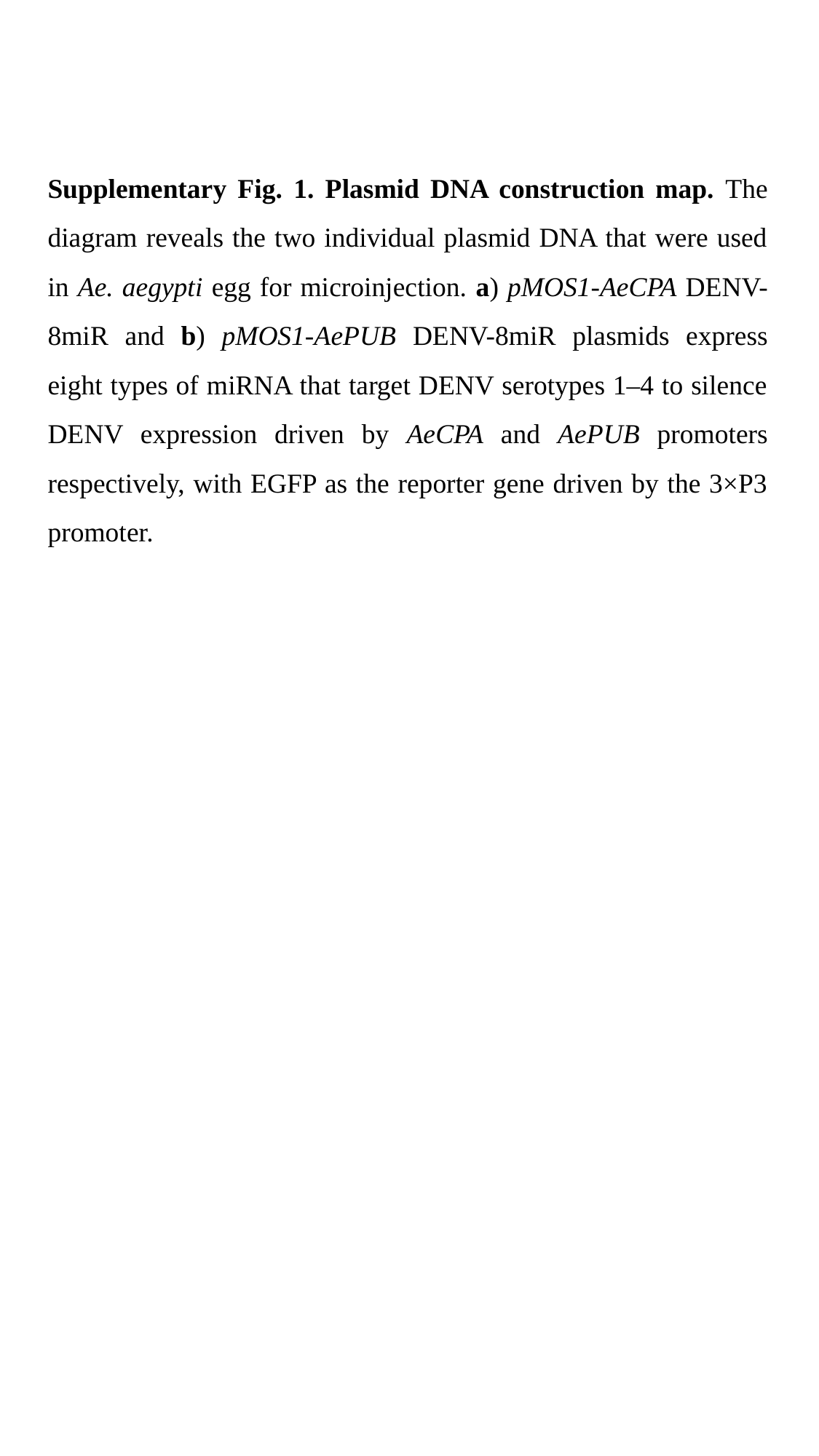

Supplementary Fig. 1. Plasmid DNA construction map. The diagram reveals the two individual plasmid DNA that were used in Ae. aegypti egg for microinjection. a) pMOS1-AeCPA DENV-8miR and b) pMOS1-AePUB DENV-8miR plasmids express eight types of miRNA that target DENV serotypes 1–4 to silence DENV expression driven by AeCPA and AePUB promoters respectively, with EGFP as the reporter gene driven by the 3×P3 promoter.

## Slide 3
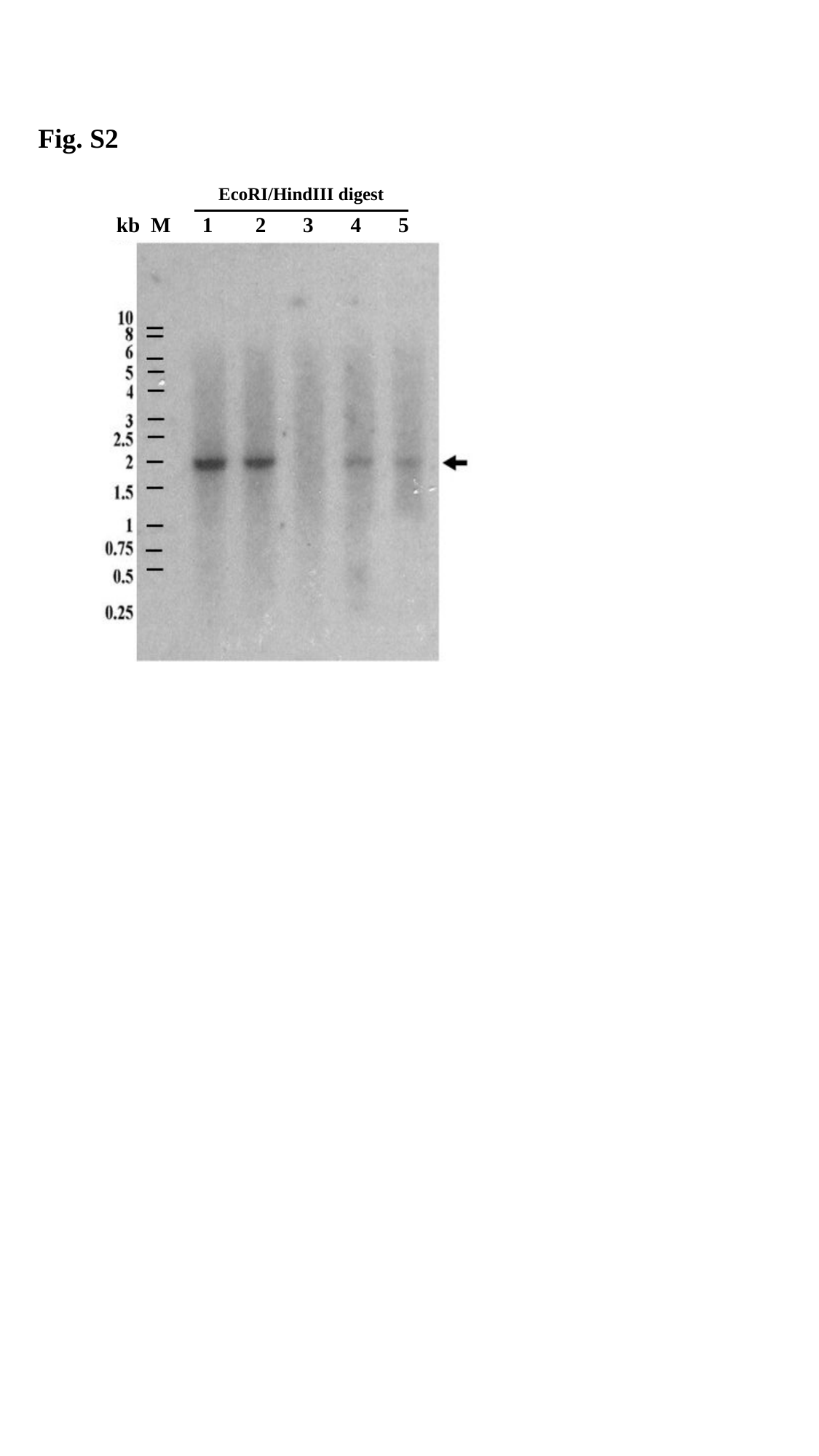

Fig. S2
EcoRI/HindIII digest
kb M 1 2 3 4 5

## Slide 4
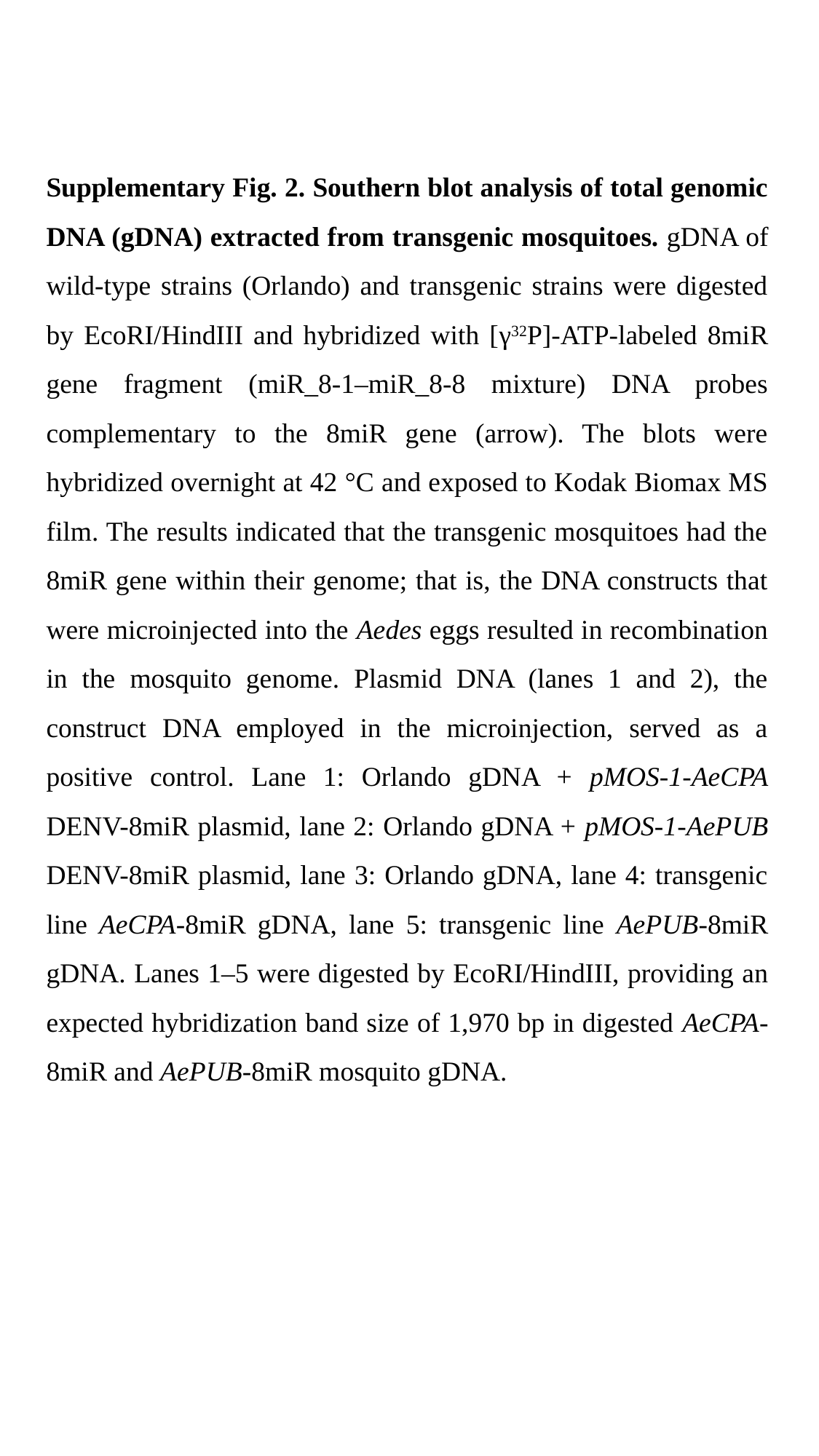

Supplementary Fig. 2. Southern blot analysis of total genomic DNA (gDNA) extracted from transgenic mosquitoes. gDNA of wild-type strains (Orlando) and transgenic strains were digested by EcoRI/HindIII and hybridized with [γ32P]-ATP-labeled 8miR gene fragment (miR_8-1–miR_8-8 mixture) DNA probes complementary to the 8miR gene (arrow). The blots were hybridized overnight at 42 °C and exposed to Kodak Biomax MS film. The results indicated that the transgenic mosquitoes had the 8miR gene within their genome; that is, the DNA constructs that were microinjected into the Aedes eggs resulted in recombination in the mosquito genome. Plasmid DNA (lanes 1 and 2), the construct DNA employed in the microinjection, served as a positive control. Lane 1: Orlando gDNA + pMOS-1-AeCPA DENV-8miR plasmid, lane 2: Orlando gDNA + pMOS-1-AePUB DENV-8miR plasmid, lane 3: Orlando gDNA, lane 4: transgenic line AeCPA-8miR gDNA, lane 5: transgenic line AePUB-8miR gDNA. Lanes 1–5 were digested by EcoRI/HindIII, providing an expected hybridization band size of 1,970 bp in digested AeCPA-8miR and AePUB-8miR mosquito gDNA.

## Slide 5
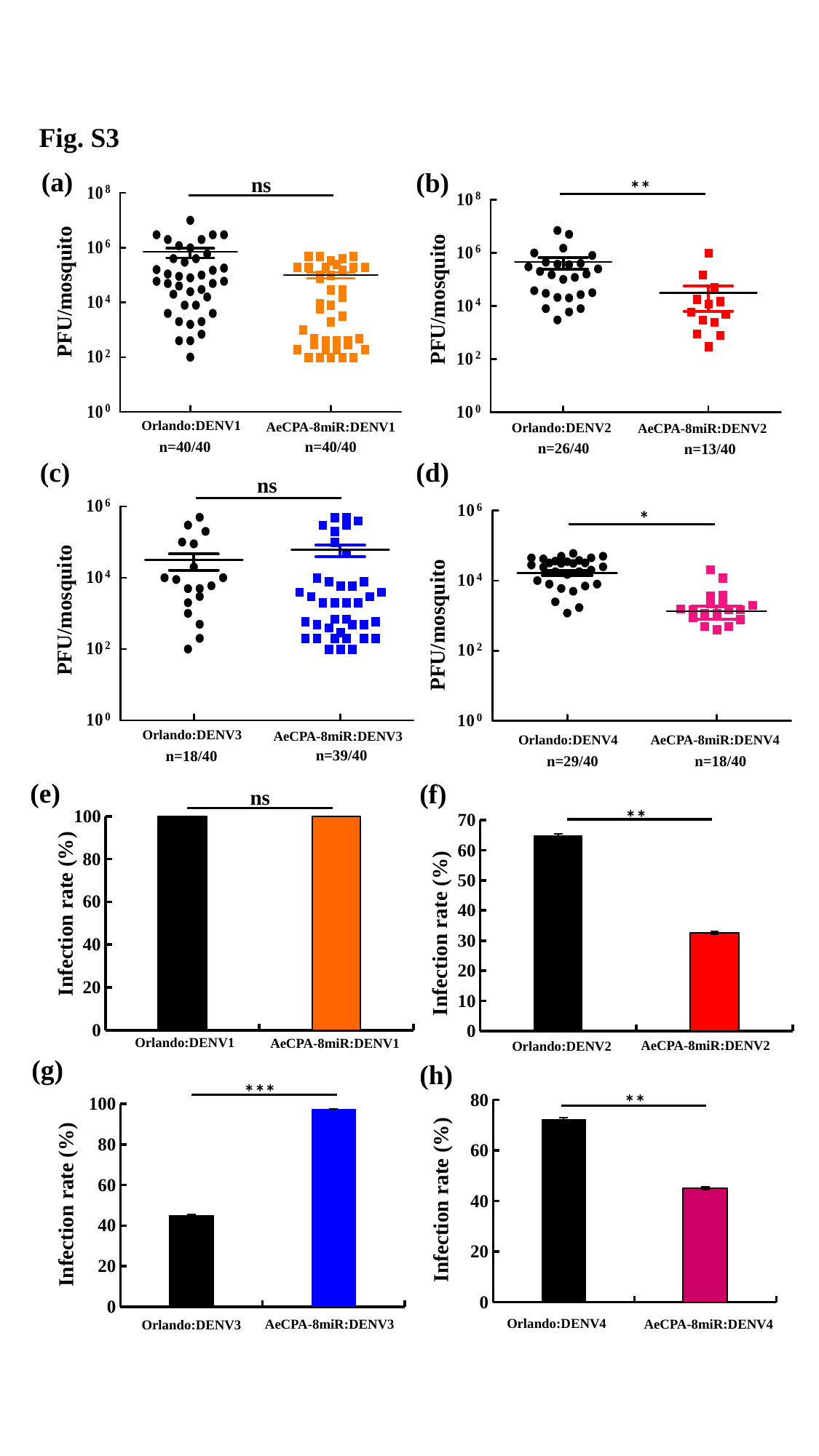

Fig. S3
(a)
(b)
**
PFU/mosquito
Orlando:DENV2
AeCPA-8miR:DENV2
n=26/40
n=13/40
ns
PFU/mosquito
Orlando:DENV1
AeCPA-8miR:DENV1
n=40/40
n=40/40
(c)
(d)
ns
*
PFU/mosquito
PFU/mosquito
Orlando:DENV3
AeCPA-8miR:DENV3
AeCPA-8miR:DENV4
Orlando:DENV4
n=39/40
n=18/40
n=18/40
n=29/40
(e)
ns
### Chart
| Category | |
|---|---|
| Orlando: DENV 1 | 100.0 |
| CPA>8MIR:DENV 1 | 100.0 |Infection rate (%)
Orlando:DENV1
AeCPA-8miR:DENV1
(f)
**
### Chart
| Category | |
|---|---|
| Orlando: DENV 2 | 65.0 |
| CPA>8MIR:DENV 2 | 32.5 |Infection rate (%)
AeCPA-8miR:DENV2
Orlando:DENV2
(g)
***
### Chart
| Category | |
|---|---|
| Orlando: DENV 3 | 45.0 |
| CPA>8MIR:DENV 3 | 97.5 |Infection rate (%)
AeCPA-8miR:DENV3
Orlando:DENV3
(h)
**
Infection rate (%)
Orlando:DENV4
AeCPA-8miR:DENV4
### Chart
| Category | |
|---|---|
| Orlando: DENV 4 | 72.5 |
| CPA>8MIR:DENV 4 | 45.0 |

## Slide 6
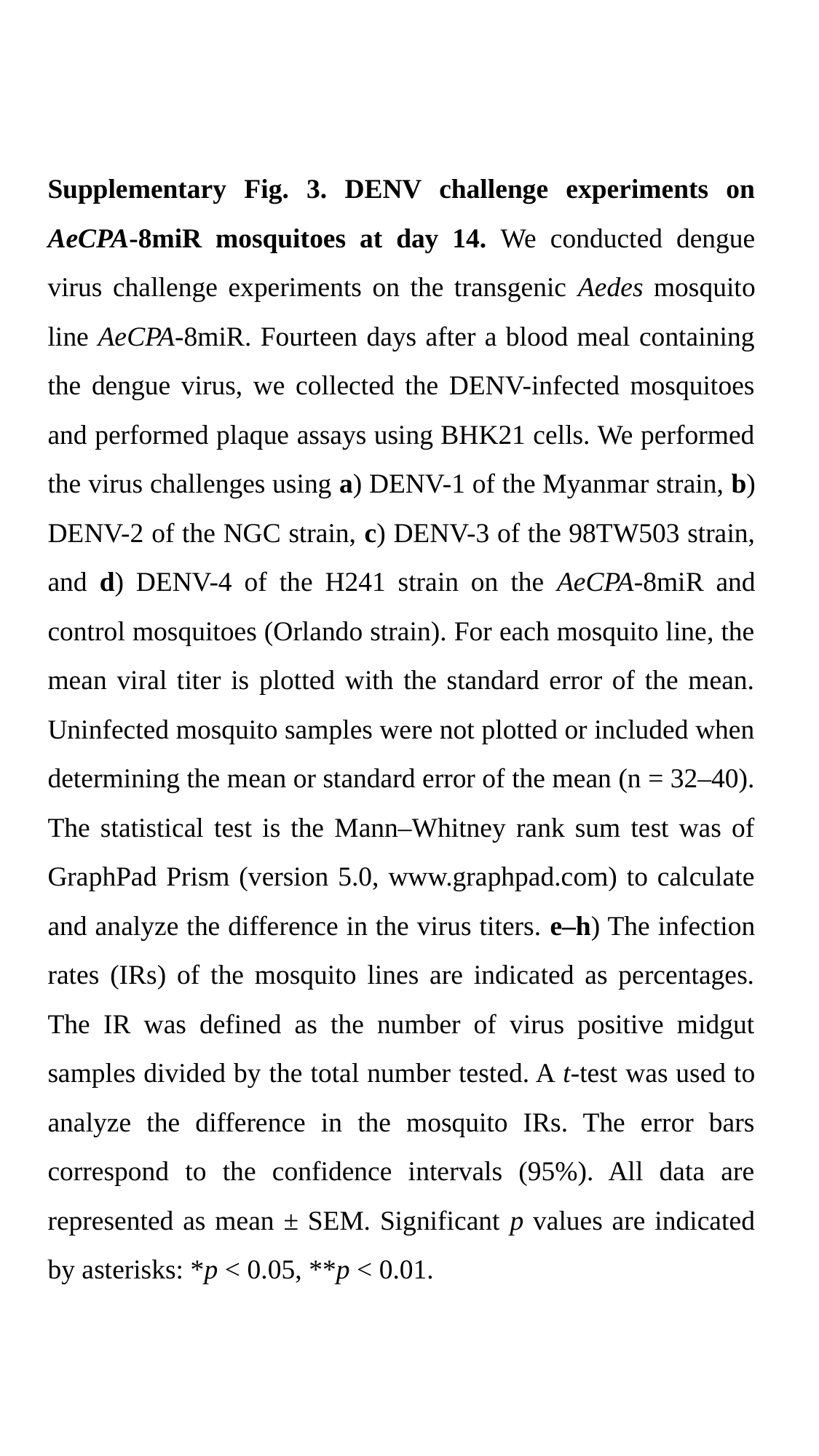

Supplementary Fig. 3. DENV challenge experiments on AeCPA-8miR mosquitoes at day 14. We conducted dengue virus challenge experiments on the transgenic Aedes mosquito line AeCPA-8miR. Fourteen days after a blood meal containing the dengue virus, we collected the DENV-infected mosquitoes and performed plaque assays using BHK21 cells. We performed the virus challenges using a) DENV-1 of the Myanmar strain, b) DENV-2 of the NGC strain, c) DENV-3 of the 98TW503 strain, and d) DENV-4 of the H241 strain on the AeCPA-8miR and control mosquitoes (Orlando strain). For each mosquito line, the mean viral titer is plotted with the standard error of the mean. Uninfected mosquito samples were not plotted or included when determining the mean or standard error of the mean (n = 32–40). The statistical test is the Mann–Whitney rank sum test was of GraphPad Prism (version 5.0, www.graphpad.com) to calculate and analyze the difference in the virus titers. e–h) The infection rates (IRs) of the mosquito lines are indicated as percentages. The IR was defined as the number of virus positive midgut samples divided by the total number tested. A t-test was used to analyze the difference in the mosquito IRs. The error bars correspond to the confidence intervals (95%). All data are represented as mean ± SEM. Significant p values are indicated by asterisks: *p < 0.05, **p < 0.01.

## Slide 7
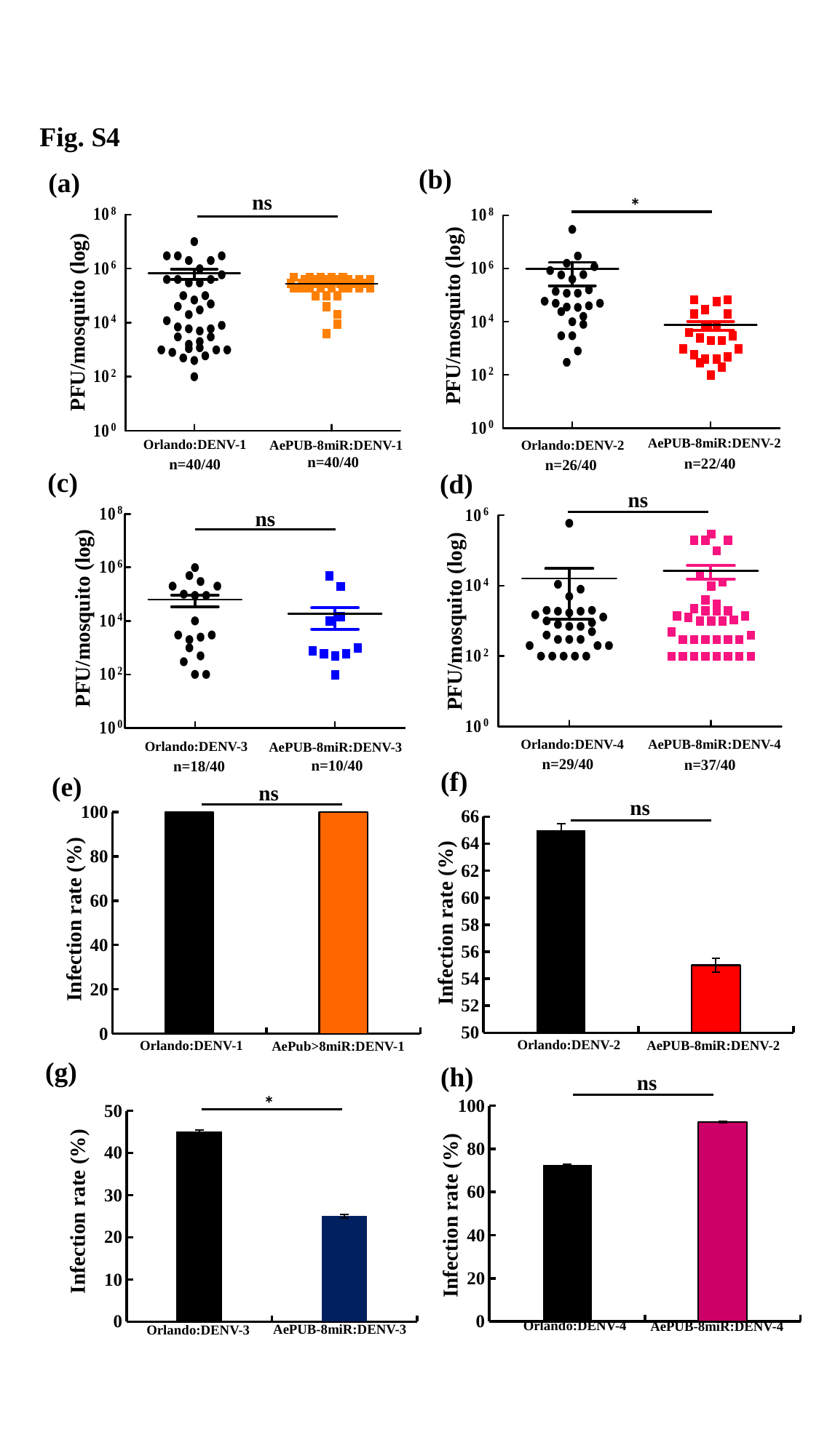

Fig. S4
(b)
*
PFU/mosquito (log)
AePUB-8miR:DENV-2
Orlando:DENV-2
n=22/40
n=26/40
(a)
ns
PFU/mosquito (log)
Orlando:DENV-1
AePUB-8miR:DENV-1
n=40/40
n=40/40
(c)
ns
PFU/mosquito (log)
Orlando:DENV-3
AePUB-8miR:DENV-3
n=10/40
n=18/40
(d)
ns
PFU/mosquito (log)
Orlando:DENV-4
AePUB-8miR:DENV-4
n=29/40
n=37/40
(f)
ns
### Chart
| Category | |
|---|---|
| Orlando: DENV 2 | 65.0 |
| CPA>8MIR:DENV 2 | 55.00000000000001 |Infection rate (%)
Orlando:DENV-2
AePUB-8miR:DENV-2
(e)
ns
### Chart
| Category | |
|---|---|
| Orlando: DENV 1 | 100.0 |
| CPA>8MIR:DENV 1 | 100.0 |Infection rate (%)
Orlando:DENV-1
AePub>8miR:DENV-1
(g)
*
### Chart
| Category | |
|---|---|
| Orlando: DENV 3 | 45.0 |
| CPA>8MIR:DENV 3 | 25.0 |Infection rate (%)
AePUB-8miR:DENV-3
Orlando:DENV-3
(h)
ns
### Chart
| Category | |
|---|---|
| Orlando: DENV 4 | 72.5 |
| CPA>8MIR:DENV 4 | 92.5 |Infection rate (%)
Orlando:DENV-4
AePUB-8miR:DENV-4

## Slide 8
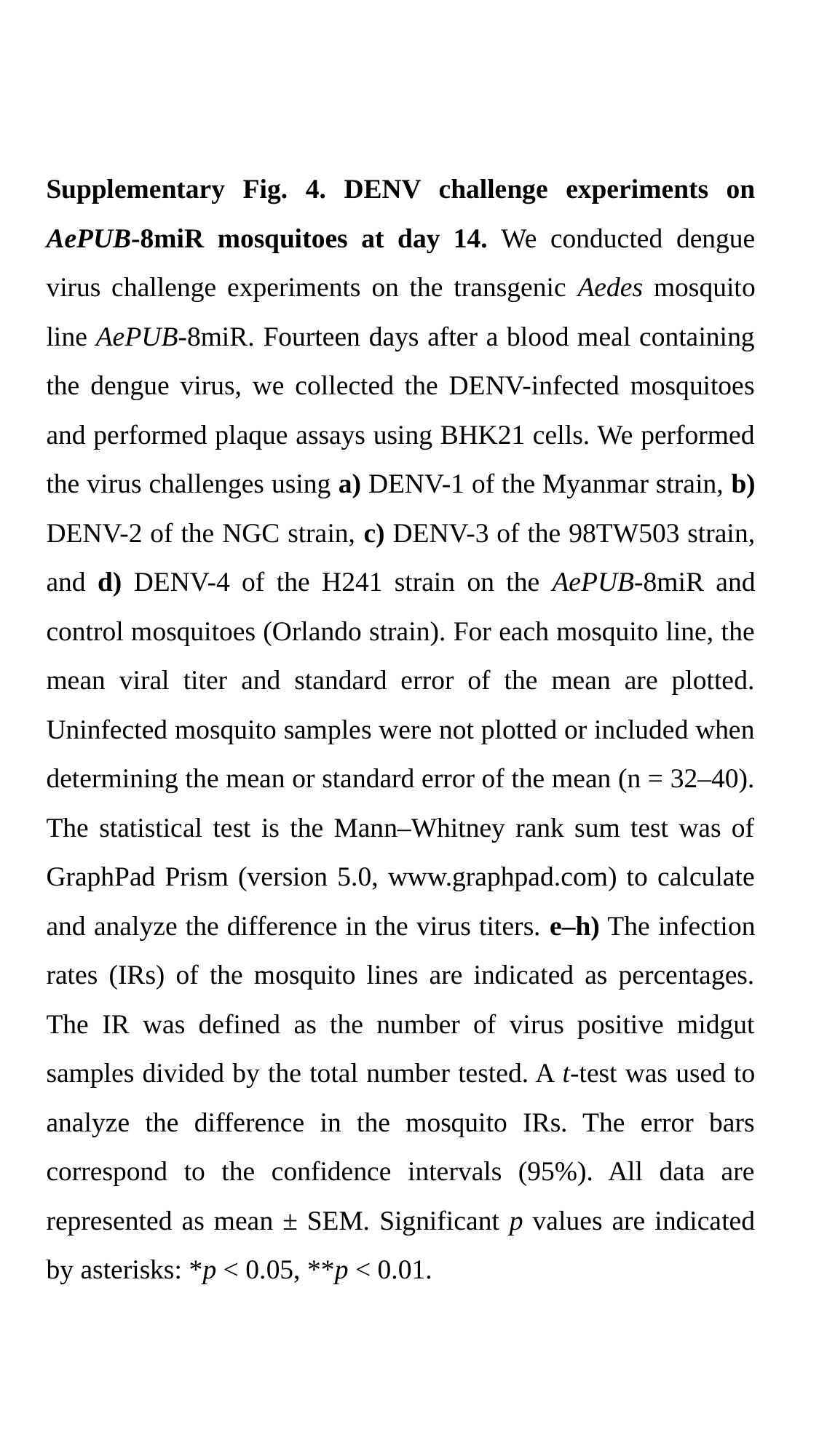

Supplementary Fig. 4. DENV challenge experiments on AePUB-8miR mosquitoes at day 14. We conducted dengue virus challenge experiments on the transgenic Aedes mosquito line AePUB-8miR. Fourteen days after a blood meal containing the dengue virus, we collected the DENV-infected mosquitoes and performed plaque assays using BHK21 cells. We performed the virus challenges using a) DENV-1 of the Myanmar strain, b) DENV-2 of the NGC strain, c) DENV-3 of the 98TW503 strain, and d) DENV-4 of the H241 strain on the AePUB-8miR and control mosquitoes (Orlando strain). For each mosquito line, the mean viral titer and standard error of the mean are plotted. Uninfected mosquito samples were not plotted or included when determining the mean or standard error of the mean (n = 32–40). The statistical test is the Mann–Whitney rank sum test was of GraphPad Prism (version 5.0, www.graphpad.com) to calculate and analyze the difference in the virus titers. e–h) The infection rates (IRs) of the mosquito lines are indicated as percentages. The IR was defined as the number of virus positive midgut samples divided by the total number tested. A t-test was used to analyze the difference in the mosquito IRs. The error bars correspond to the confidence intervals (95%). All data are represented as mean ± SEM. Significant p values are indicated by asterisks: *p < 0.05, **p < 0.01.

## Slide 9
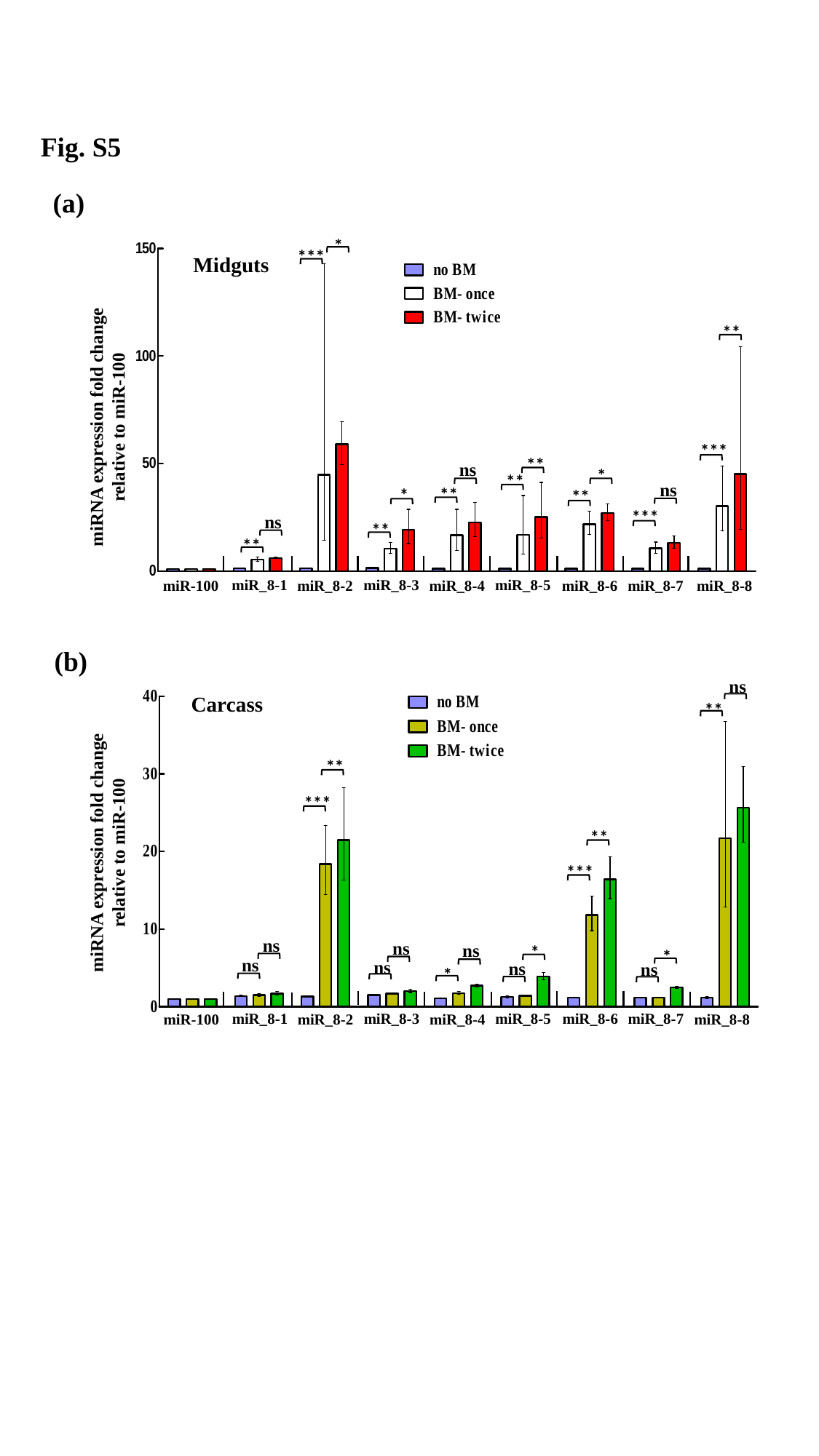

Fig. S5
(a)
*
***
Midguts
**
miRNA expression fold change relative to miR-100
***
**
ns
*
**
ns
**
*
**
***
ns
**
**
miR_8-3
miR_8-5
miR_8-1
miR_8-6
miR_8-7
miR_8-4
miR_8-8
miR_8-2
miR-100
(b)
ns
Carcass
**
**
***
**
miRNA expression fold change relative to miR-100
***
ns
ns
ns
*
*
ns
ns
ns
ns
*
miR_8-3
miR_8-5
miR_8-1
miR_8-6
miR_8-7
miR_8-4
miR_8-8
miR_8-2
miR-100

## Slide 10
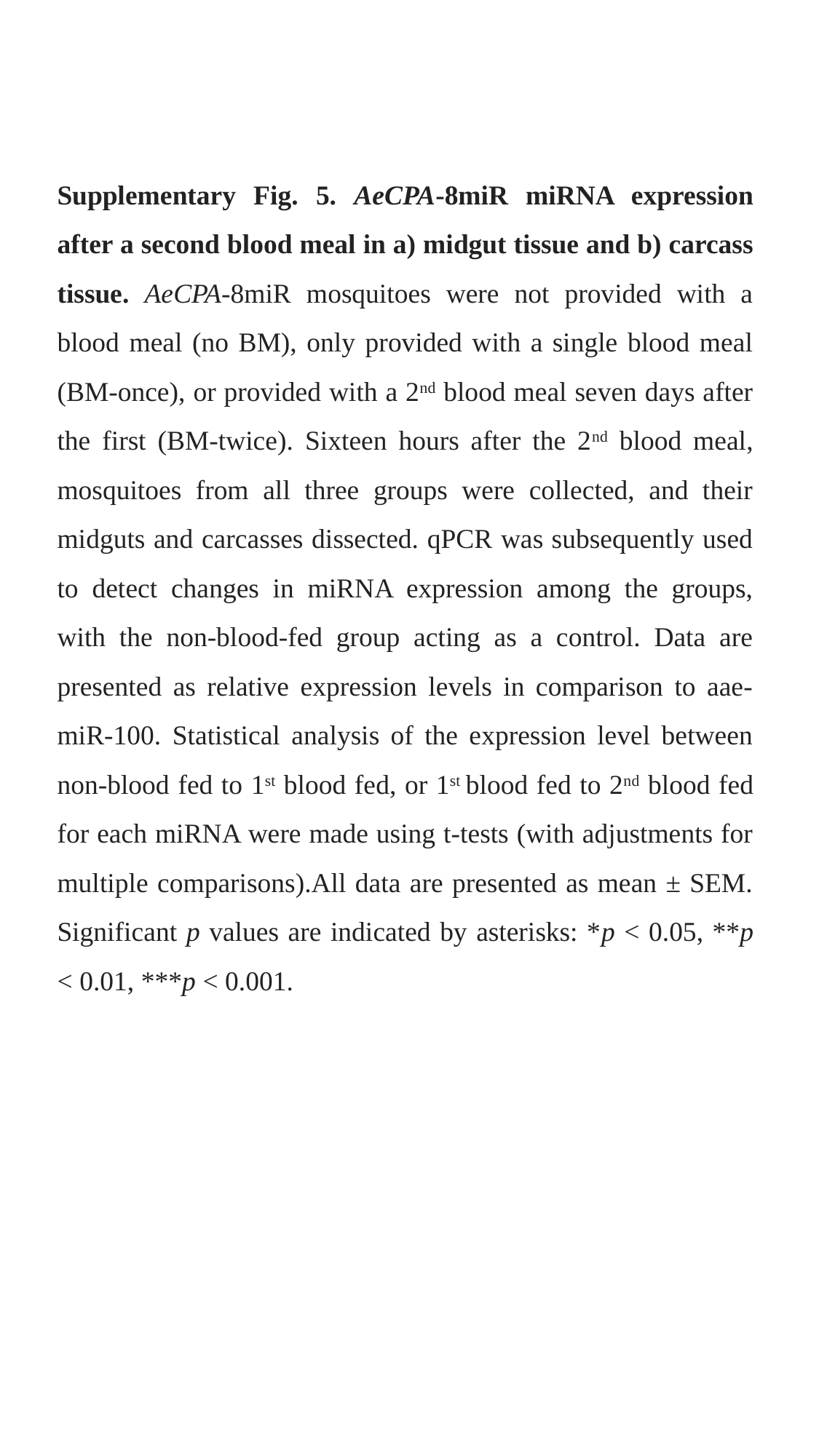

Supplementary Fig. 5. AeCPA-8miR miRNA expression after a second blood meal in a) midgut tissue and b) carcass tissue. AeCPA-8miR mosquitoes were not provided with a blood meal (no BM), only provided with a single blood meal (BM-once), or provided with a 2nd blood meal seven days after the first (BM-twice). Sixteen hours after the 2nd blood meal, mosquitoes from all three groups were collected, and their midguts and carcasses dissected. qPCR was subsequently used to detect changes in miRNA expression among the groups, with the non-blood-fed group acting as a control. Data are presented as relative expression levels in comparison to aae-miR-100. Statistical analysis of the expression level between non-blood fed to 1st blood fed, or 1st blood fed to 2nd blood fed for each miRNA were made using t-tests (with adjustments for multiple comparisons).All data are presented as mean ± SEM. Significant p values are indicated by asterisks: *p < 0.05, **p < 0.01, ***p < 0.001.

## Slide 11
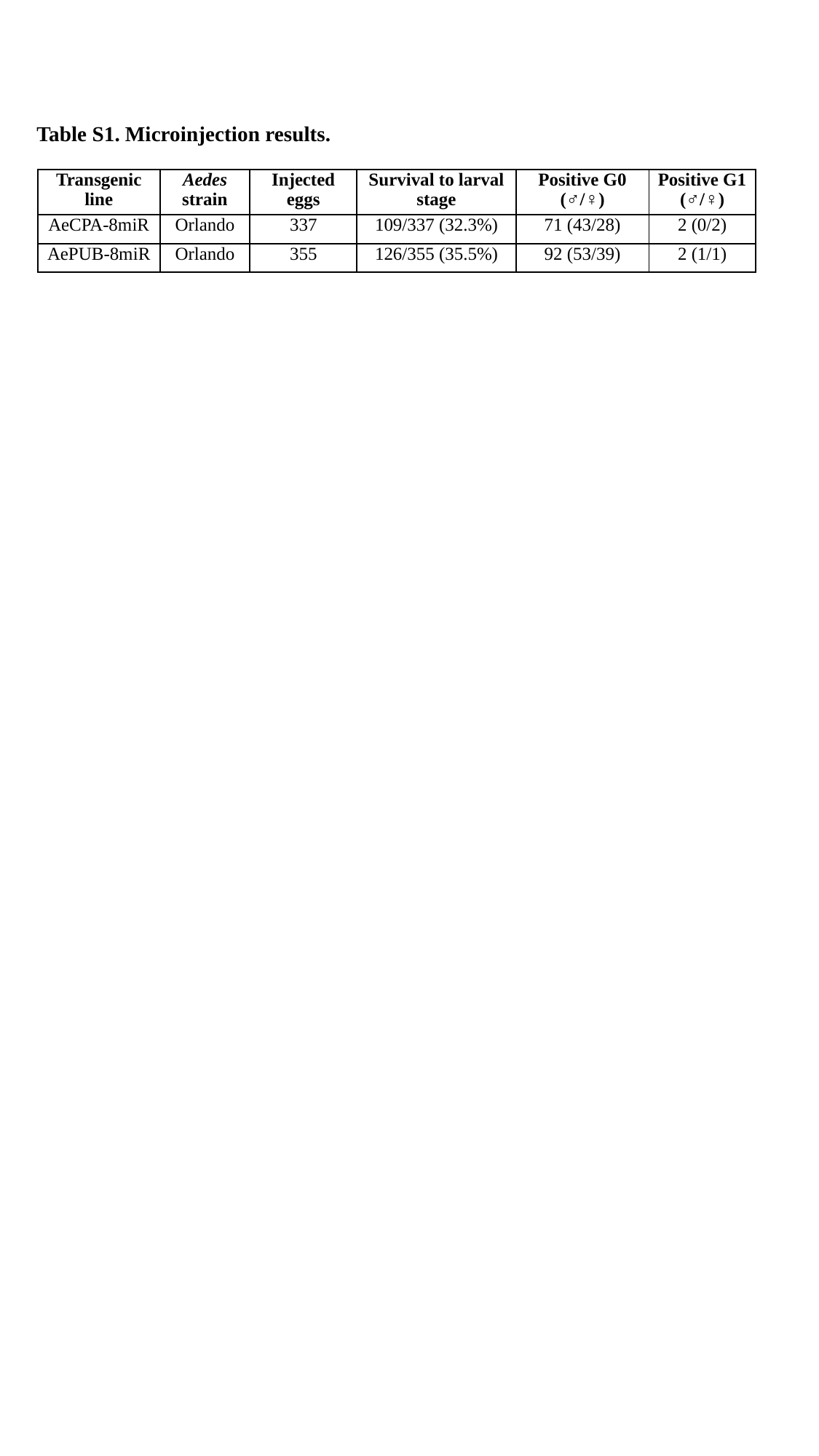

Table S1. Microinjection results.
| Transgenic line | Aedes strain | Injected eggs | Survival to larval stage | Positive G0 (♂/♀) | Positive G1 (♂/♀) |
| --- | --- | --- | --- | --- | --- |
| AeCPA-8miR | Orlando | 337 | 109/337 (32.3%) | 71 (43/28) | 2 (0/2) |
| AePUB-8miR | Orlando | 355 | 126/355 (35.5%) | 92 (53/39) | 2 (1/1) |

## Slide 12
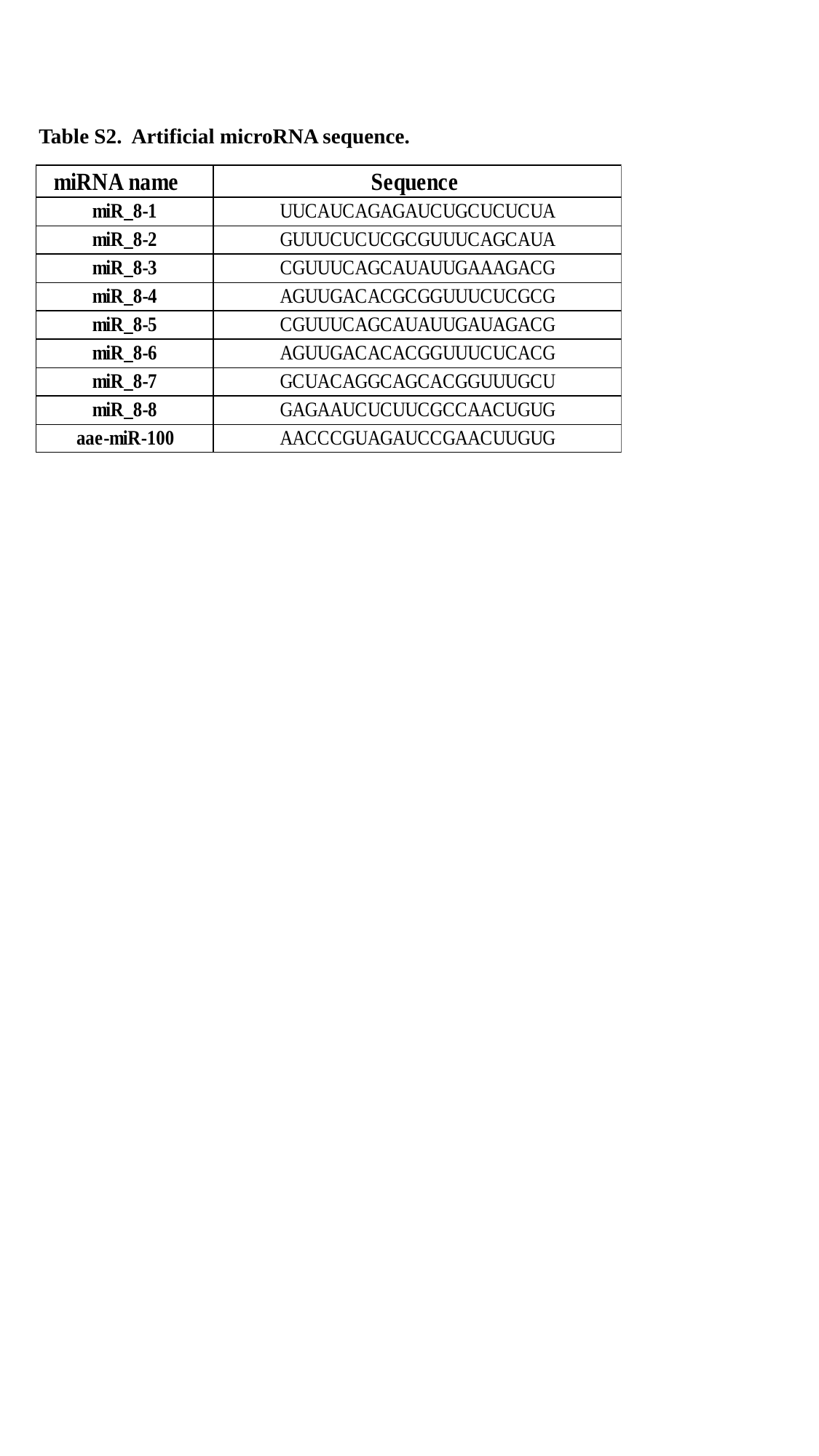

Table S2. Artificial microRNA sequence.

## Slide 13
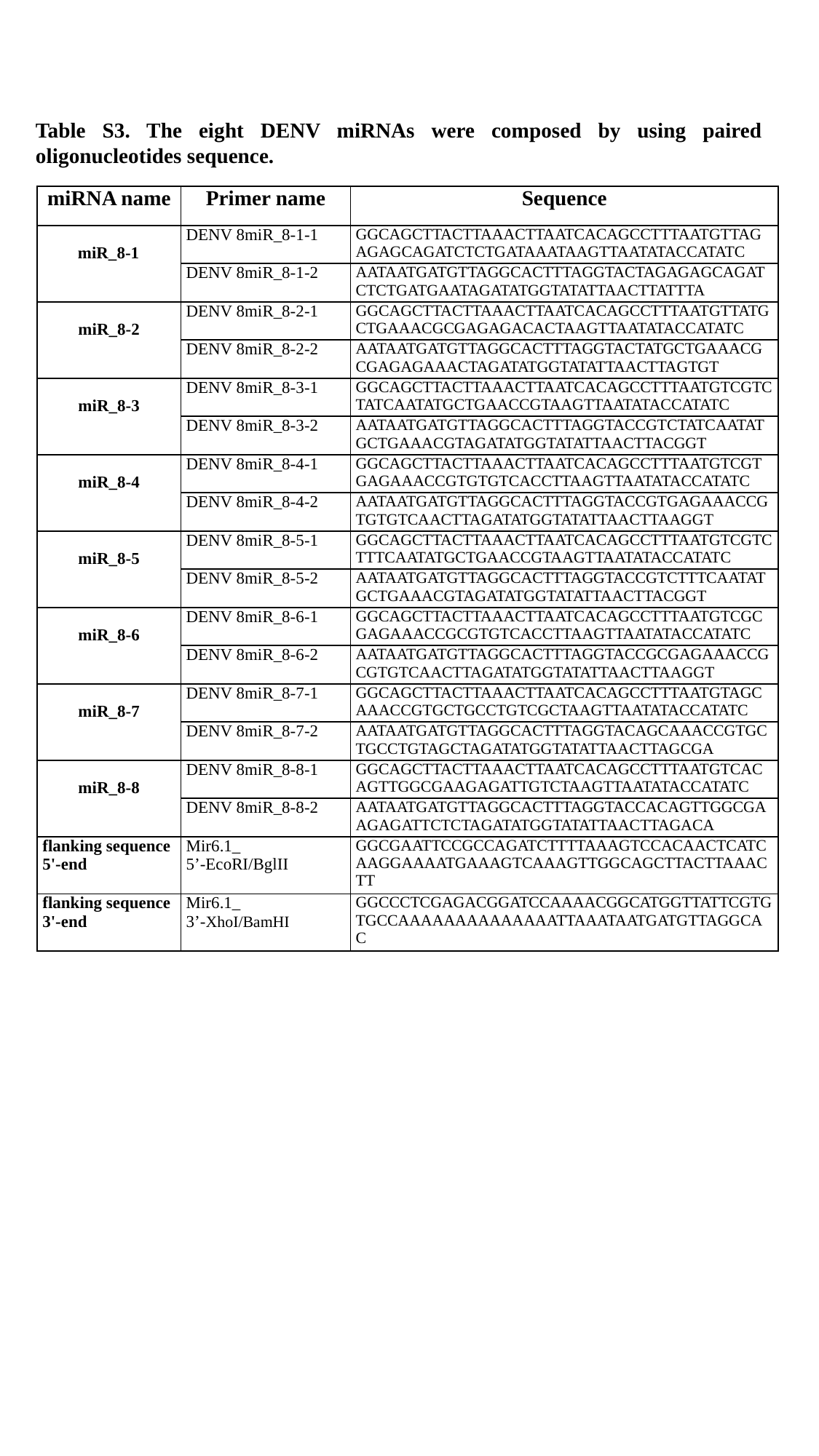

Table S3. The eight DENV miRNAs were composed by using paired oligonucleotides sequence.
| miRNA name | Primer name | Sequence |
| --- | --- | --- |
| miR\_8-1 | DENV 8miR\_8-1-1 | GGCAGCTTACTTAAACTTAATCACAGCCTTTAATGTTAGAGAGCAGATCTCTGATAAATAAGTTAATATACCATATC |
| | DENV 8miR\_8-1-2 | AATAATGATGTTAGGCACTTTAGGTACTAGAGAGCAGATCTCTGATGAATAGATATGGTATATTAACTTATTTA |
| miR\_8-2 | DENV 8miR\_8-2-1 | GGCAGCTTACTTAAACTTAATCACAGCCTTTAATGTTATGCTGAAACGCGAGAGACACTAAGTTAATATACCATATC |
| | DENV 8miR\_8-2-2 | AATAATGATGTTAGGCACTTTAGGTACTATGCTGAAACGCGAGAGAAACTAGATATGGTATATTAACTTAGTGT |
| miR\_8-3 | DENV 8miR\_8-3-1 | GGCAGCTTACTTAAACTTAATCACAGCCTTTAATGTCGTCTATCAATATGCTGAACCGTAAGTTAATATACCATATC |
| | DENV 8miR\_8-3-2 | AATAATGATGTTAGGCACTTTAGGTACCGTCTATCAATATGCTGAAACGTAGATATGGTATATTAACTTACGGT |
| miR\_8-4 | DENV 8miR\_8-4-1 | GGCAGCTTACTTAAACTTAATCACAGCCTTTAATGTCGTGAGAAACCGTGTGTCACCTTAAGTTAATATACCATATC |
| | DENV 8miR\_8-4-2 | AATAATGATGTTAGGCACTTTAGGTACCGTGAGAAACCGTGTGTCAACTTAGATATGGTATATTAACTTAAGGT |
| miR\_8-5 | DENV 8miR\_8-5-1 | GGCAGCTTACTTAAACTTAATCACAGCCTTTAATGTCGTCTTTCAATATGCTGAACCGTAAGTTAATATACCATATC |
| | DENV 8miR\_8-5-2 | AATAATGATGTTAGGCACTTTAGGTACCGTCTTTCAATATGCTGAAACGTAGATATGGTATATTAACTTACGGT |
| miR\_8-6 | DENV 8miR\_8-6-1 | GGCAGCTTACTTAAACTTAATCACAGCCTTTAATGTCGCGAGAAACCGCGTGTCACCTTAAGTTAATATACCATATC |
| | DENV 8miR\_8-6-2 | AATAATGATGTTAGGCACTTTAGGTACCGCGAGAAACCGCGTGTCAACTTAGATATGGTATATTAACTTAAGGT |
| miR\_8-7 | DENV 8miR\_8-7-1 | GGCAGCTTACTTAAACTTAATCACAGCCTTTAATGTAGCAAACCGTGCTGCCTGTCGCTAAGTTAATATACCATATC |
| | DENV 8miR\_8-7-2 | AATAATGATGTTAGGCACTTTAGGTACAGCAAACCGTGCTGCCTGTAGCTAGATATGGTATATTAACTTAGCGA |
| miR\_8-8 | DENV 8miR\_8-8-1 | GGCAGCTTACTTAAACTTAATCACAGCCTTTAATGTCACAGTTGGCGAAGAGATTGTCTAAGTTAATATACCATATC |
| | DENV 8miR\_8-8-2 | AATAATGATGTTAGGCACTTTAGGTACCACAGTTGGCGAAGAGATTCTCTAGATATGGTATATTAACTTAGACA |
| flanking sequence 5'-end | Mir6.1\_ 5’-EcoRI/BglII | GGCGAATTCCGCCAGATCTTTTAAAGTCCACAACTCATCAAGGAAAATGAAAGTCAAAGTTGGCAGCTTACTTAAACTT |
| flanking sequence 3'-end | Mir6.1\_ 3’-XhoI/BamHI | GGCCCTCGAGACGGATCCAAAACGGCATGGTTATTCGTGTGCCAAAAAAAAAAAAAATTAAATAATGATGTTAGGCAC |

## Slide 14
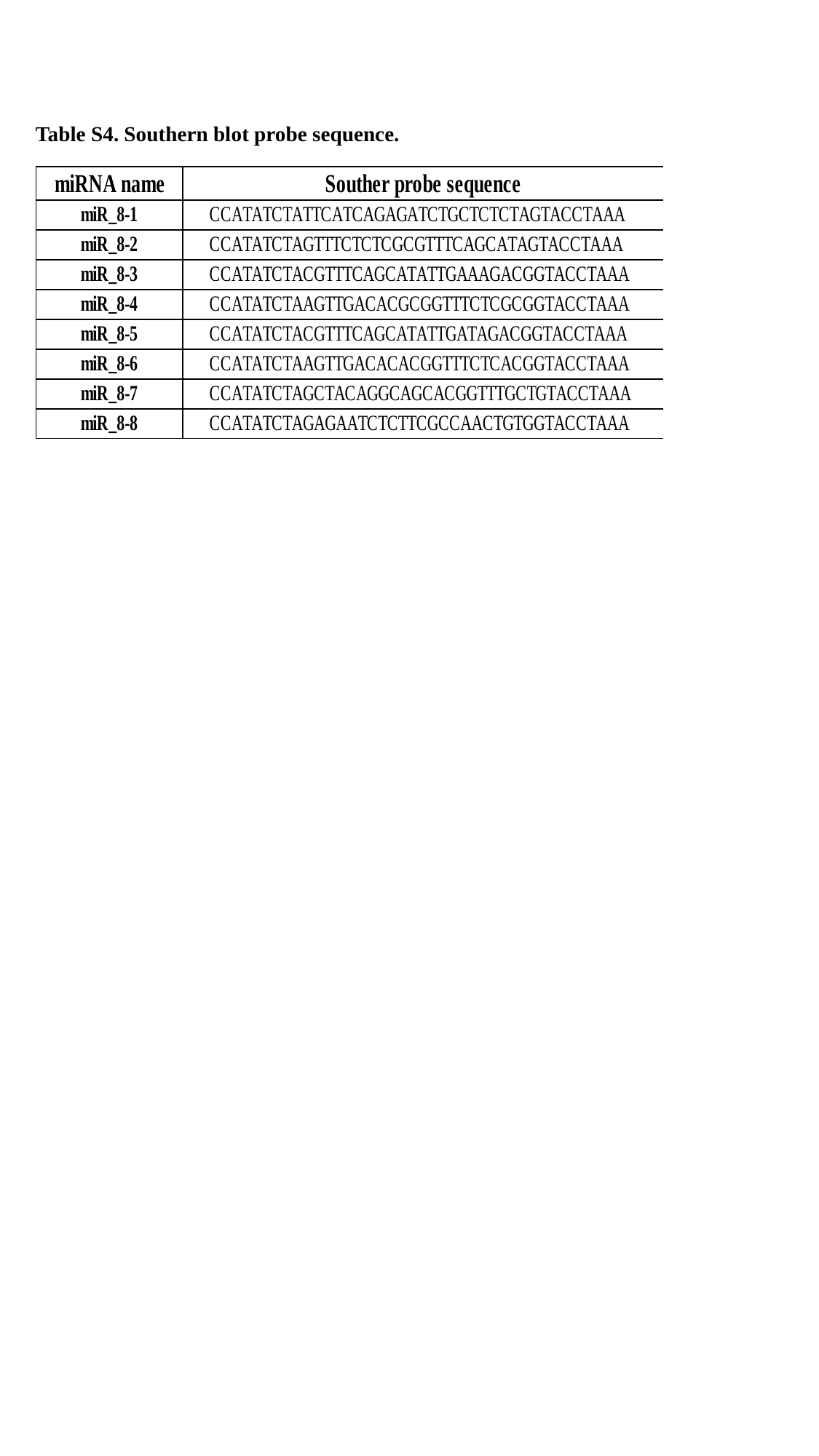

Table S4. Southern blot probe sequence.

## Slide 15
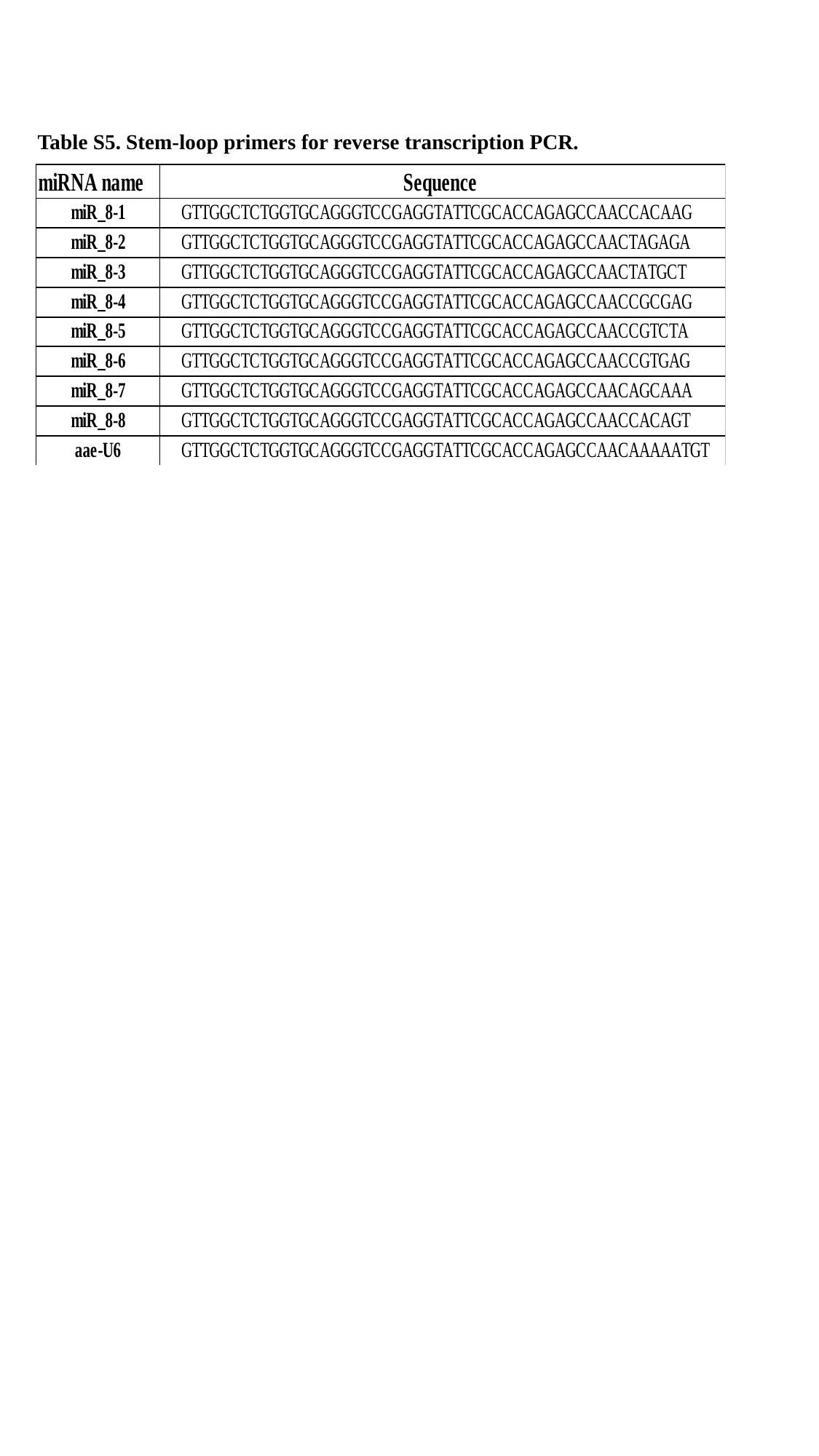

Table S5. Stem-loop primers for reverse transcription PCR.

## Slide 16
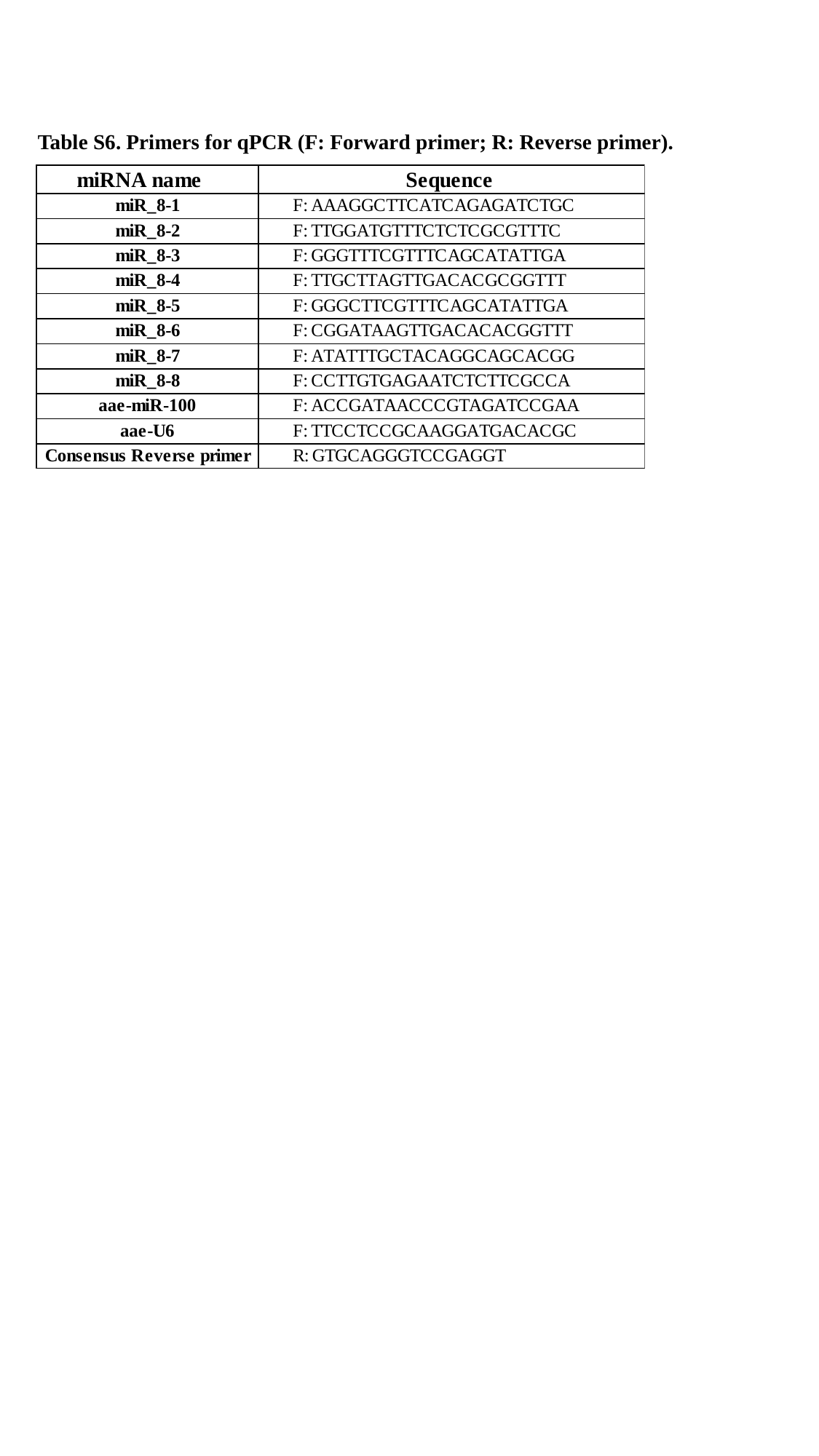

Table S6. Primers for qPCR (F: Forward primer; R: Reverse primer).
